# Morphological screening of mesenchymal mammary tumor organoids to identify drugs that reverse epithelial-mesenchymal transition

**DOI:** 10.1038/s41467-021-24545-3

**Published:** 2021-07-12

**Authors:** Na Zhao, Reid T. Powell, Xueying Yuan, Goeun Bae, Kevin P. Roarty, Fabio Stossi, Martina Strempfl, Michael J. Toneff, Hannah L. Johnson, Sendurai A. Mani, Philip Jones, Clifford C. Stephan, Jeffrey M. Rosen

**Affiliations:** 1 Department of Molecular and Cellular Biology, Baylor College of Medicine, Houston, TX USA; 2 Center for Translational Cancer Research, Texas A&M Health Science Center, Institute of Biosciences and Technology, Houston, TX USA; 3 Integrated Microscopy Core, Baylor College of Medicine, Houston, TX USA; 4 Graz University of Technology, NAWI Graz, Graz, Styria Austria; 5 Department of Biology, Widener University, Chester, PA USA; 6 Department of Translational Molecular Pathology, University of Texas MD Anderson Cancer Center, Houston, TX USA; 7 Institute of Applied Cancer Science (IACS), University of Texas MD Anderson Cancer Center, Houston, TX USA

**Keywords:** Biological models, Breast cancer

## Abstract

The epithelial-mesenchymal transition (EMT) has been implicated in conferring stem cell properties and therapeutic resistance to cancer cells. Therefore, identification of drugs that can reprogram EMT may provide new therapeutic strategies. Here, we report that cells derived from claudin-low mammary tumors, a mesenchymal subtype of triple-negative breast cancer, exhibit a distinctive organoid structure with extended “spikes” in 3D matrices. Upon a miR-200 induced mesenchymal-epithelial transition (MET), the organoids switch to a smoother round morphology. Based on these observations, we developed a morphological screening method with accompanying analytical pipelines that leverage deep neural networks and nearest neighborhood classification to screen for EMT-reversing drugs. Through screening of a targeted epigenetic drug library, we identified multiple class I HDAC inhibitors and Bromodomain inhibitors that reverse EMT. These data support the use of morphological screening of mesenchymal mammary tumor organoids as a platform to identify drugs that reverse EMT.

## Abstract (web summary)

The epithelial-mesenchymal transition (EMT) has been implicated in stem cell properties and therapeutic resistance in cancer. Here, the authors show organoids derived from mesenchymal breast cancers exhibit a spikey structure which can be reverted and exploited for screening drugs that reverse EMT.

## Introduction

Triple-negative breast cancer (TNBC) is a heterogeneous group of tumors defined by the lack of expression of estrogen receptor (ER), progesterone receptor (PR), and Her2. TNBC has been classified into minimally four subtypes, among which the claudin-low subtype is the most mesenchymal, and patients with these tumors have high rates of metastases and chemoresistance^1–4^. The epithelial–mesenchymal transition (EMT) is an evolutionarily conserved developmental program during which cells lose epithelial markers and gain mesenchymal traits. In response to pleiotropic signals, a group of EMT-associated transcription factors and epigenetic regulators orchestrate this complex transition. EMT confers metastatic properties to cancer cells by enhancing motility, invasion, and resistance to apoptotic stimuli. Moreover, intermediate or “partial EMT” tumor cells acquire increased plasticity, display cancer stem cell properties, and exhibit marked therapeutic resistance^5–7^. An EMT signature was also found to be associated with a poor-prognosis subtype of breast cancer defined by a distinct tumor immune microenvironment^8^. Previous neoadjuvant clinical trials revealed that residual breast cancers after conventional endocrine therapy (letrozole) or chemotherapy (docetaxel) displayed these intermediate EMT features as well as tumor-initiating properties^9^. Thus, the EMT state is often the “default” phenotype observed during therapeutic resistance across breast cancer subtypes. In a preclinical claudin-low/mesenchymal genetically modified mouse (GEM) model developed in our laboratory, re-expression of the miR-200 microRNAs, which are master regulators of the mesenchymal-epithelial transition (MET)^10–12^, reversed cancer stem cell properties and sensitized tumors to chemotherapy^13^. Therefore, identification of drugs that can phenocopy miR-200 and reprogram cells into an epithelial-like state may provide new therapeutic strategies.

During the past years, several efforts have been made to screen for EMT-reversing drugs. We previously developed the “Z-cad” dual lentiviral fluorescent reporter system which comprises destabilized green fluorescent protein (GFP) containing the *ZEB1* 3′ UTR and red fluorescent protein (RFP) driven by the E-cadherin (*CDH1*) promoter^14^. Employing this reporter system, we carried out a high-throughput small molecule drug screening and identified several GSK3β inhibitors capable of inhibiting EMT in MDA-MB-231 cells in two-dimensional (2D) culture^15^. Similarly, other studies utilized a *CDH1* promoter-driven luciferase reporter in high-throughput screening and found that protein kinase A (PKA) activation and histone deacetylase (HDAC) inhibitors were able to restore epithelial differentiation^16,17^.

Recently, three-dimensional (3D) organoid culture systems have emerged as improved models for cell behavior analysis and personalized medicine applications. Organoids maintain cell–cell interactions, display more similarities to the original tissues with respect to their histology and transcriptomics, and provide more accurate predictions for therapeutic responses^18–25^. To date, dozens of drug screens have been carried out in organoid cultures which primarily use viability-based endpoints in the determination of drug activity^21,26–32^. Since reversing EMT does not necessarily alter cell viability, and many mesenchymal cells display unique organoid morphology as shown in this study, the development of a morphological screening platform focusing on EMT reprogramming provides valuable toolsets for drug discovery.

Over the past decade, revolutions of computer vision have resulted in the development of a fundamentally different type of image analysis based on deep neural network (dNN) architectures. These systems work directly on the raw images and provide an integrated approach that combines image feature extraction, typically achieved using stacked convolutional and pooling layers, with classification tasks using artificial neural network^33–36^. In the context of phenotypic screening, the most common applications of machine learning are retrieval of compounds that mimic a control (classification) or placing compounds with similar mechanisms of action together (clustering), which have both been successfully addressed using dNN and image embedding based approaches^37–40^. In this study, we utilized primary cell lines derived from *Trp53*-null claudin-low GEM models which generated spiky organoid structures in Matrigel. We then optimized a 3D organoid culture platform and employed an inducible miR-200c overexpression system as a control to reverse EMT. This produced two distinct morphologies which could be visually distinguished but were difficult to objectively segment, thus precluded the utilization of conventional image analysis approaches that rely on accurate segmentation. Therefore, we developed a screening approach that combines generic image features from a pre-trained dNN with a k-nearest neighbors (k-NN) model to map the similarity of experimental treatments to reference controls. Using this approach, we identified a number of small molecule inhibitors that reversed EMT in an epigenetic drug screen. These compounds fell into two broad categories, inhibitors of class I HDAC and Bromodomain (BRD). These data support the use of mesenchymal organoid morphological screens for EMT-reversing drug discovery. Such screens also serve as a first step to provide insights into the epigenetic regulation of the EMT phenotype.

## Results

### Reprogramming EMT by inducing miR-200c expression changes organoid morphology

**Fig. 1:**
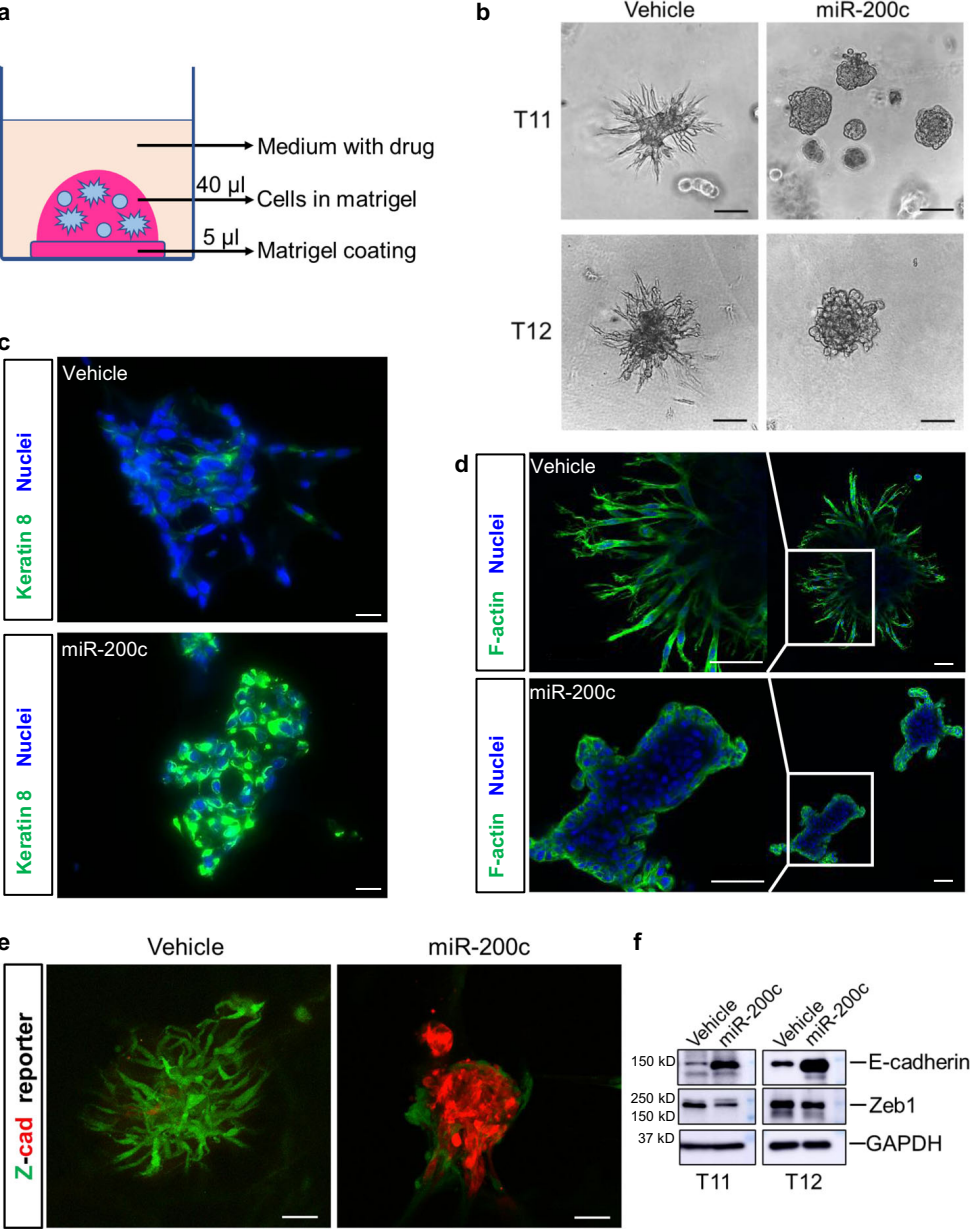
Reversing EMT by inducing miR-200c expression changes organoid morphology. **a** Schematic diagram of 3D organoid cultures in regular culture format. **b** Morphology of vehicle- or dox-treated T11 and T12 organoids by phase-contrast microscopy. Representative images of independent biological duplicates (*n* = 10). Scale bar: 100 μm. **c** IF staining of Keratin 8 in vehicle- or dox-treated T11 organoid sections. Representative images of independent biological triplicates. Scale bar: 20 μm. **d** F-actin staining of vehicle- or dox-treated T11 whole-mount organoids. Representative images of independent biological triplicates. Scale bar: 50 μm. **e** Z-cad reporter imaging in vehicle- or dox-treated T11 whole-mount organoids. EMT cells would be green and MET cells would be red based on the design of the reporter system. Representative images of independent biological triplicates. Scale bar: 50 μm. **f** Immunoblotting assay of EMT markers in vehicle- or dox-treated T11 and T12 organoids. Representative images of independent biological triplicates.

We first established several primary cell lines from *Trp53*-null murine mammary tumor models. These *Trp53*-null GEM tumors were previously established by transplantation of donor mammary epithelium from BALB/c mice, where *Trp53* was deleted from the germline, into syngeneic hosts for derivation of a variety of *Trp53*-null mammary tumors^41,42^. Comparative oncogenomics and gene profiling demonstrated that the resulting mammary tumors are representative of the corresponding human breast cancer subtypes^43–45^. As the engineered *Trp53* cassette contains neomycin resistance marker^41,46^, G418 can be added to culture medium to remove stromal cells for the generation of primary cell lines. Intriguingly, when embedded in Matrigel, single cells from T11 and T12, two *Trp53*-null claudin-low mammary tumor models, grew into organoid structures with projections invading the surrounding matrix (Fig. 1a, b). The inducible expression of the MET promoter miR-200c by addition of doxycycline (dox) to TetOn-miR-200c cells prevented the formation of these invasive protrusions without obviously affecting organoid size (Fig. 1b, Supplementary Fig. 1a, b, and Supplementary Movie 1), and markedly induced the expression of luminal cytokeratin K8 at both RNA and protein levels (Fig. 1c and Supplementary Fig. 1c–e). Phalloidin-stained F-actin revealed that cells in the claudin-low organoids were invasive as F-actin was present in structures resembling invadopodia; whereas in miR-200c induced organoids, the F-actin had a cortical alignment without evident invadopodia (Fig. 1d, Supplementary Fig. 2a, and Supplementary Movie 2–5). Moreover, miR-200c induction promoted the expression of the epithelial marker E-cadherin and reduced the expression of the mesenchymal transcription factor Zeb1, as demonstrated by Z-cad reporter analysis, immunoblotting, and immunostaining (Fig. 1e, f, and Supplementary Fig. 2b–d). This morphological switch (spiky to round) provides a unique real-time readout for EMT/MET dynamics and affords the possibility of using these 3D organoids in image-based screening to identify small molecules that can alter the EMT/MET plasticity.

### Optimization of a 3D organoid screening assay and image analysis

**Fig. 2:**
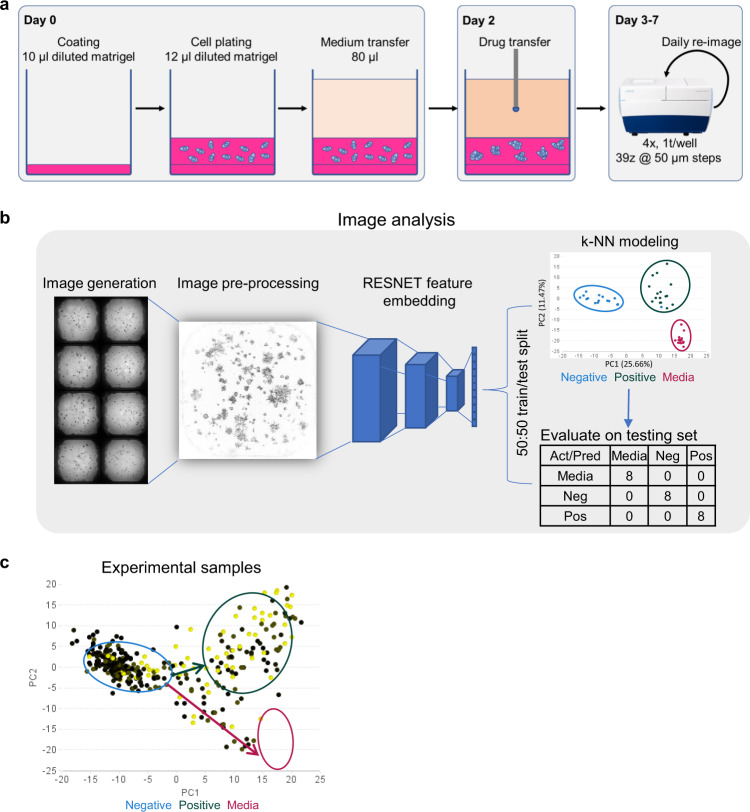
3D organoid cultures and the development of RESNET. **a** Schematic diagram of organoid cultures and screening workflow. Plates are generated by first depositing a buffer layer of 60% Matrigel to prevent cells from adhering to the bottom. Cell-containing 50% Matrigel is then added followed by media. Drugs are transferred using a 100 nl pin tool 2 days after cell seeding and serial bright-field imaging is performed in the following days. **b** Image analysis pipeline. 3D image stacks are first background corrected and projected into a single z-plane. High-level image features are then derived from the flattening layer of the RESNET-18 pre-trained on the ImageNet dataset. Principal component analysis is then used to explore response patterns (the percent of variance that is explained by each principal component is labeled to the axis) and k-nearest neighbor model is constructed for subsequent classification tasks. Assay quality is monitored using a 50/50 train/test split for each control category, which is shown in the confusion matrix. Negative (Neg), DMSO-treated wells; Positive (Pos), miR-200c induced wells; Media, cell-free wells. Act/Pred, Actual vs Predicted. **c** Principal component analysis of RESNET-18 features for experimental samples with control regions annotated (blue, negative region; dark green, positive region; red, media region). Increasing drug concentrations are shown from low (black) to high (yellow).

In order to perform screening, we first optimized an automated method to reproducibly generate organoids in a 384-well plate format. Since undiluted Matrigel is too viscous for robotic liquid transfer, we tested organoid growth in 50% Matrigel diluted with medium and confirmed that the organoid phenotypes were similar to those observed in undiluted Matrigel (Supplementary Fig. 3a). For organoid cultures, a cell-free coating of 60% Matrigel was added to the bottom of wells, which prevented the adherence of cells to the bottom of the well (Supplementary Fig. 3b). Next, cells diluted in 50% Matrigel were transferred to pre-coated plates, resulting in an ~1-mm thick layer at the meniscus which contained 500 cells per well. After allowing cells to recover for 2 days, plates were treated with drugs using a pin-transfer (Fig. 2a). Each plate contained 16 vehicle (DMSO)-treated negative control wells, 16 dox-treated wells (for miR-200c induction) as an EMT-reversing positive control, and 16 cell-free Media-only wells as a toxicity control. Cells were incubated in the presence of drug for a duration of 5 days, after which bright-field z-stack images were collected using the ImageXpress Micro Confocal system.

Next, we developed an analytical workflow to map visually similar wells (phenomimetics) to reference control wells using an image embedding approach. Here we used an 18-layer residual neural networks (RESNET-18) architecture^47^ that was pre-trained on the ImageNet challenge dataset^34^ and extracted information at the flattening layer in-between the convolutional and artificial neural network layers to effectively convert images into a descriptive numeric vector. We then performed either principal component analysis (PCA) or k-NN clustering to visualize the phenotypic landscape and map experimental wells to control-like conditions, respectively. The resulting PCA showed that the controls were well clustered and that the positive and negative controls are primarily separated in the first principal component, while the positive and media-only control are separated by the second principal component. This subsequently prompted us to generate a k-NN model to statistically map drug-treated wells to the reference controls, respectively (Fig. 2b). In the initial optimization assays, time-lapse microscopy was carried out to identify a time point where the negative (DMSO) and positive (dox) controls could be fully statistically resolved. We found that DMSO-treated and miR-200 induced wells are fully distinguishable after 5 days of drug treatment, and this time point was used in all subsequent screening assays (Supplementary Fig. 3c). To monitor assay quality, we sub-sampled the control wells into non-overlapping training and testing sets. All testing wells can be correctly assigned to their respective treatment groups using this analytical pipeline, implying that the model is functional (Fig. 2b). In order to evaluate the effects of subsampling and the number of neighbors used for classification, we trained one hundred iterations of k-NN models using randomly assigned subsamples for each K-value between 1 and 6. Those data showed minimal differences when trained on any given subsample or K-values, indicating that this method is robust and not influenced by the sampling method (Supplementary Fig. 4).

### Identification of EMT-reversing epigenetic small molecule inhibitors through mesenchymal organoid morphological screening

Multiple studies show that significant epigenetic changes are associated with EMT and are frequently required to mediate the functions of EMT transcription factors^48,49^. Therefore, screening for EMT-reversing epigenetic therapies should provide fundamental insights into the epigenetic basis of EMT and may lead to novel therapeutic approaches. We obtained an epigenetic drug library comprising 65 small molecule chemicals targeting multiple epigenetic modifiers (readers, writers, and erasers) from the Institute for Applied Cancer Science (IACS) at MD Anderson Cancer Center (Supplementary Data 1). These compounds were screened at four concentrations with two independent biological replicates for both T11 and T12 organoid cultures. Exploratory PCA analysis showed that drug-treated wells resided within the component space of controls and showed some assemblance of dose-dependency with lower concentration of drugs primarily in the negative control region and higher concentrations migrating toward the positive and media-only controls (Fig. 2c). These data encouraged us to train a series of k-NN models using plate level and batch aggregated datasets. The models generated from each assay plate are used to provide a real-time feedback of the assay robustness as well as to qualify individual experiments for inclusion into the larger dataset. The batch aggregated models are iteratively retrained as qualified assay batches are added and are ultimately the model that is used to classify experimental drugs. For all models, half of the controls were used as the training set, while the remainder were used for statistical evaluation of accuracy calculated from the confusion matrix. From these data, we can see that the resulting k-NN models generally had a high accuracy when trained at the batch (80%) and cumulative (90%) levels for both the T11 and T12 cell models, suggesting that the assay is robust with well-resolved controls and there are no major batch effects present (Supplementary Fig. 4).

**Fig. 3:**
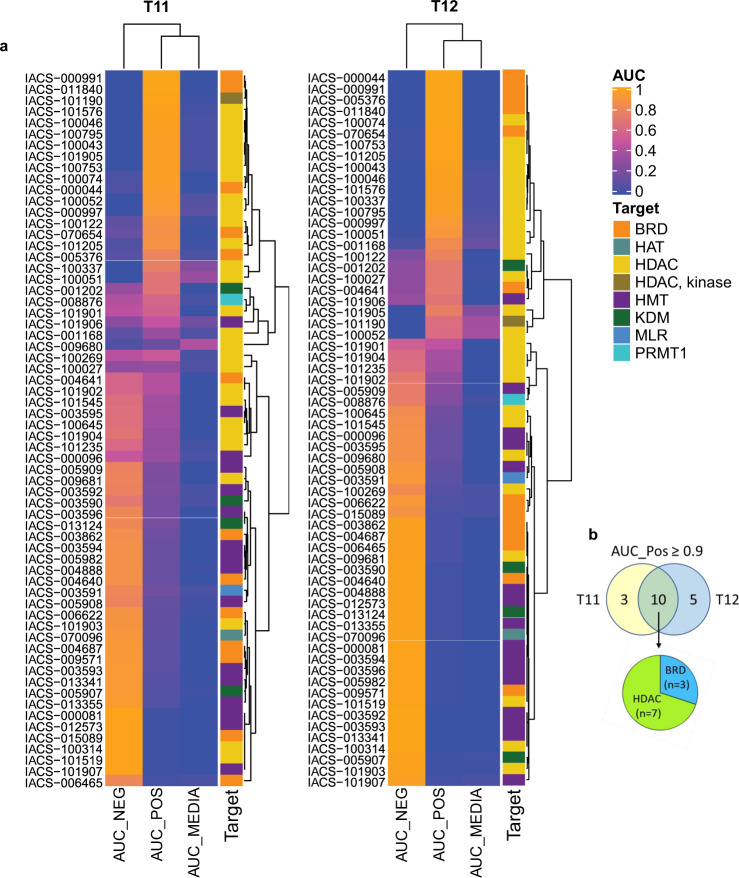
EMT-reversing epigenetic drug screening in organoid cultures. **a** Clustered heatmap of IACS epigenetic drug library screening in T11 and T12 organoids. Drug-treated organoids were assigned a continuous ranking of drug activity (represented by area under the dose–response curve, AUC) for each of the three mapped categories: NEG (no effect, DMSO-like), POS (reduced branching structures, miR-200c induction-like), or MEDIA (no organoid present). Support vector regression was used to fit probability of each class across concentrations, and clustering was done based on these values. The known molecular targets for each drug are shown in the annotation column. The description of each IACS drug is shown in Supplementary Data 1. **b** Venn diagram showing overlapping top positive-like drug hits and their target categories in T11 and T12 models. BRD bromodomain, HAT histone acetyltransferase, HDAC histone deacetylase, HMT histone methyltransferases, KDM histone lysine demethylase, MLR methyl-lysine reader, PRMT1 protein arginine methyltransferase 1.

Next, drug-treated organoids were assigned a continuous ranking of drug activity (represented by Area Under the dose–response Curve, AUC) using the classification probabilities for each of the three mapped categories namely: Negative (no effect, DMSO-like), Positive (reduced branching structures, miR-200c induction-like), or Media (no organoid present). In our dataset, we observed a number of drugs with saturated signal (i.e., drugs where all data points are either positive-like or negative-like), drugs that followed a sigmoidal dose–response curve, and finally drugs with a multi-stage response where positive-like effects switch to toxic effects (i.e., resembled Media-only wells) as a function of increasing drug concentrations (Supplementary Data 2). Therefore, to provide a robust curve fit across all of the observed response patterns, we chose to use a support vector regression-based method. This approach does not make any assumptions on the underlying shape of the curve and is therefore more generalizable when compared to fitting data against a 4-parameter logistic regression curve^50^. In both T11 and T12 models, HDAC and BRD inhibitors were retrieved as classes of drugs that consistently resembled miR-200 induction, as shown by their high AUC_Pos score (Fig. 3a and Supplementary Data 2). The extensive overlap between the top hits in both claudin-low organoid models suggests that HDACs and BRD proteins are common regulators of EMT (Fig. 3b). Inhibitors that target histone methyltransferases (HMT, including PRMT1/3/4/5, G9a, DOT1L, SETD7, EZH2), histone lysine demethylase (KDM, including LSD1, KDM4A/5B/6B), or methyl-lysine reader (MLR, including L3MBTL3) were more similar to DMSO-treated control wells across the majority of concentrations.

### Validation of top EMT-reversing hits

**Fig. 4:**
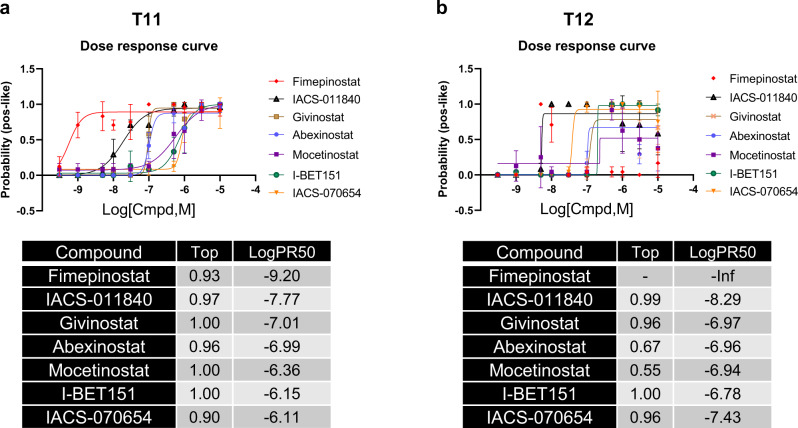
Dose curve responses of the top drug hits. Dose–response curves of the probability resembling the positive control by concentration for T11 (**a**) and T12 (**b**) organoids. Data points represent the mean and standard deviation of four technical replicates. Curves are fit using a constrained 4-parameter logistic using Graphpad Prism. The resulting curve fit parameters describing efficacy (top probability score) and potency (LogPR50 concentration) are summarized in the lower table.

To validate the primary organoid screening, we conducted more complete dose–response assays of the top-ranked positive control-like hits. For these assays, each drug was tested against 10 concentrations in half-log steps with technical quadruplicates in both T11 and T12 models. The quality control analyses showed 100% performance in discriminating control wells (Supplementary Fig. 4). In the dose–response curves, all the drugs were validated with consistent results in the overlapping concentration ranges when compared to the primary screen (Fig. 4a, b). Importantly, the concentration of drug that is required to switch the organoid phenotype was resolved from this set of experiments. Accordingly, drug activity was ranked on the basis of potency, defined here as the concentration of drug that is required to have a 50% probability of belonging to the positive control-like classification (PR_50_). The most potent EMT-reversing drug in T11 was Fimepinostat (IACS-101190), a dual HDAC and PI3K inhibitor, which displayed strong positive-like effect at nanomolar concentrations (Fig. 4a). In the T12 model, Fimepinostat showed a positive-like effect at 10 nM ranges but switched to a more toxic like phenotype at higher concentrations, resulting in an atypical curve that failed to converge (Fig. 4b). HDAC inhibitors (Givinostat, Abexinostat, and Mocetinostat) showed reprogramming effects at near micromolar concentrations (Fig. 4b). To evaluate the potential drug effects on cell viability, we performed a counter screen at the end time point using 3D Cell TiterGlo (CTG) that measures ATP-levels as a surrogate of cell viability. These data show that the concentration of most drugs required to shift organoids from resembling the negative to the positive controls was less than that causing significant toxicity (Supplementary Fig. 5), indicative of a reprogramming event.

**Fig. 5:**
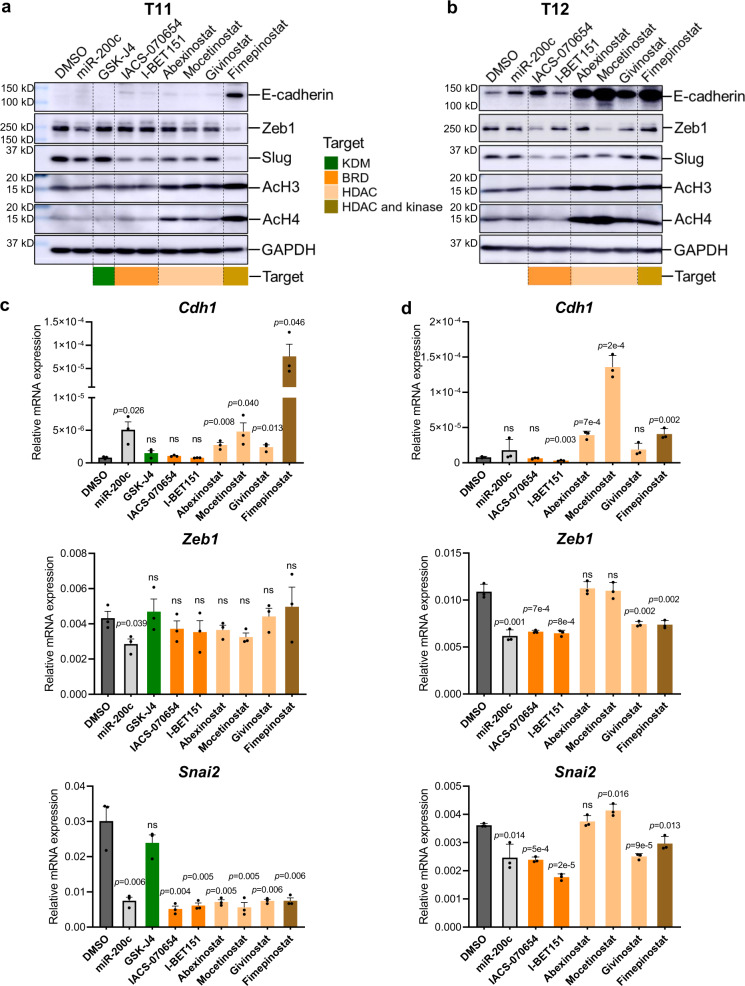
Inhibitors targeting class I HDAC and BRD proteins reverse EMT. **a** Immunoblotting assay of EMT markers in treated T11 cells in 2D culture. Fimepinostat was applied at 200 nM and all other drugs were applied at 1 μM concentration. Representative images of independent biological triplicates. **b** Immunoblotting assay of EMT markers in treated T12 cells in 2D culture. Fimepinostat was applied at 20 nM and all other drugs were applied at 1 μM concentration. Representative images of independent biological triplicates. KDM histone lysine demethylase, BRD bromodomain, HDAC histone deacetylase. **c** qPCR of EMT marker genes in treated T11 cells. Drug concentrations as described in (**a**). Data are presented relative to *Actb* and shown as mean ± s.e.m. of independent biological triplicates. **d** qPCR of EMT marker genes in treated T12 cells. Drug concentrations as described in (**b**). Data are presented relative to *Actb* and shown as mean ± s.e.m. of independent biological triplicates. ns, not significant. Statistical analysis by unpaired Student *t*-tests (two-tailed).

To further confirm the effects of the top hits on EMT reprogramming, we conducted immunoblotting assays in drug-treated T11 and T12 cells in 2D cultures. Upon Fimepinostat treatment, we observed a potent induction of E-cadherin in T11 and T12 and conversely a marked reduction of Zeb1 and Slug expression in T11 cells (Fig. 5a, b). Moreover, Fimepinostat treatment-induced *CDH1* promoter-driven RFP reporter activity and *Cdh1* RNA expression in a dose-dependent manner (Supplementary Fig. 6a, b), indicating the transcriptional activation of E-cadherin by Fimepinostat. The two BRD inhibitors, IACS-070654 and I-BET151 (IACS-000044), reduced Slug protein and RNA expression (Fig. 5a–d). The three class I HDAC inhibitors, Abexinostat (IACS-100046), Mocetinostat (IACS-100074), and Givinostat (IACS-100753), were able to reduce Slug expression in T11 cells and induce E-cadherin expression in T12 cells. These three HDAC inhibitors also increased *CDH1*-RFP reporter activity and *Cdh*1 RNA expression in both T11 and T12 cells, indicating a transcription regulation (Fig. 5a–d, and Supplementary Fig. 6c). All these HDAC inhibitors were effective at upregulating the acetylation levels of histone H3 and H4, indicating that the drugs hit their targets. For comparison, GSK-J4 (IACS-001202), which is a KDM6B inhibitor that did not show up as the top positive hit in the screen, failed to cause detectable changes in EMT proteins or RNA (Fig. 5a, c). Collectively, using two independent organoid models, these studies illustrate the potential application of morphological screening of claudin-low organoids in the identification of EMT-reprogramming drugs with high accuracy and sensitivity.

**Fig. 6:**
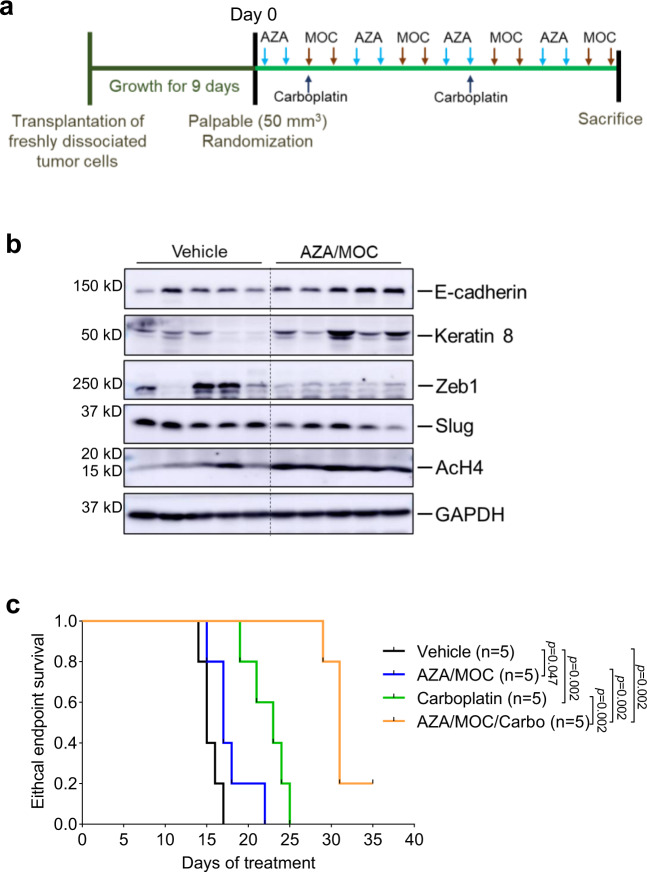
Mocetinostat in combination with Azacytidine sensitizes mesenchymal tumors to chemotherapy. **a** Schematic of treatment schedule. Treatment is initiated when tumors are palpable. Mice are treated with Azacytidine (AZA) and Mocetinostat (MOC) in alternating 2-day cycles to avoid drug interference. Carboplatin is injected weekly. **b** Immunoblotting assay of T12 tumor tissues harvested at the ethical endpoint from mice that were treated with either vehicle alone or epigenetic therapies. *n* = 5 for each group. **c** Kaplan–Meier survival curve of T12 tumor-bearing mice from treatment start time (Day 0). The log-rank test (two-tailed) was used to test for the significant differences of Kaplan–Meier survival curves between groups. *n* = 5 for each group.

To further evaluate whether reprogramming of EMT can increase chemosensitivity, we transplanted T12 tumor cells into the mammary fat pad of mice and started drug treatment when tumors become palpable (Fig. 6a). Since Mocetinostat (MOC) is currently under clinical trials in several tumor types and showed good reprogramming efficacy in T12, we decided to test its effect in vivo. It has been reported that HDAC inhibitors often work synergistically with DNA methyltransferase inhibitors such as Azacytidine (AZA) in reactivation of aberrantly silenced genes in cancer^51^; thus, we included AZA with MOC for further studies. Of note, Azacytidine alone did not alter organoid morphology nor did it induce E-cadherin expression in T12 cells (Supplementary Fig. 7). When transplanted in vivo, T12 tumors treated with combined AZA and MOC exhibited EMT reversal although some variation was observed among tumors from different mice as expected since these immunoblots were performed on end-stage tumors containing both stroma and immune cells (Fig. 6b and Supplementary Fig. 8). T12 tumors are very aggressive and reached the ethical endpoint 2 weeks after treatment initiation. Epigenetic therapy or chemotherapy alone prolonged mice survival by a few days. However, the treatment of MOC and AZA sensitized T12 tumors to carboplatin, indicated by the prolonged survival of tumor-bearing mice (Fig. 6c). These data suggest that drugs identified from the morphological organoid screen potentially may be utilized for combination therapies in vivo.

## Discussion

Most breast cancer deaths in the United States can be ascribed to recurrent metastatic disease that is resistant to conventional therapies. Exploring the molecular mechanisms and developing new therapies for treatment resistance is imperative especially for TNBC for which there is no targeted therapy. As EMT confers cancer stemness and chemoresistance, the discovery of drugs that alter the EMT program may provide new therapeutic approaches to sensitize breast cancers to standard-of-care chemotherapy and to overcome therapy resistance. Moreover, the impact of EMT-reversing drug discovery may extend well beyond resistant TNBC to other types of therapy-resistant cancer and metastatic disease. A unique feature of our organoid screen is that it can differentiate EMT-reprogramming effects from direct cell killing, which is the endpoint in most cell viability-based assays. This is critical because these targeted therapies may ultimately be used in combination with standard-of-care chemotherapy in the clinic.

Tumor organoids are being increasingly utilized for basic and translation research. However, many of the endpoints used to detect drug actions rely on biochemical readouts. At the time of this publication, other groups have successfully implemented single organoid morphometric analysis for drug screening applications^52–54^. One favorable aspect of using image-based analysis is that multiple aspects of biological response can be simultaneously captured and subsequently used to identify unique molecular mechanisms of action^52,55,56^. In these studies, distinct morphological patterns were identified by image segmentation. Unlike those studies, our model system creates a highly invasive and often-interconnecting morphology that confounded segmentation. For this reason, we generate an analytical approach using dNN-derived image features that does not rely on explicit segmentation to provide an automated screening solution for this model system. Both image classification and regression can be performed using dNN inspired approaches. Indeed, multiple methods have been proposed to implement dNN driven analysis, which includes training dNN architectures on de novo data, fixing potions of the dNN architecture and fine-tuning the remainder, and directly using the features produced from pre-trained convolutional layers as an input for secondary statistical modeling. From a functional standpoint, training an entire deep neural network takes hundreds to many thousands of representative images that are subjectively determined as a function of the complexity of images, number of categories being trained, and specific structure of the dNN. Importantly, the heavy requirement of utilizing large data is primarily driven by the training of convolutional layers responsible for feature extraction. However, it has been observed that convolutional layers trained on very large visually diverse images sets, such as the ImageNet dataset, are generally informative and can be “fine-tuned” to novel applications by supplementing de novo datasets^57^. In turn, this effectively reduces the demand for large volumes of data and time required to train a network and is often referred to as transfer learning. Finally, image embedding simply applies the pre-trained convolutional layers to images and outputs a descriptive numeric vector extracted from various layers of the architecture. This approach skips fine-tuning and can be effectively used to provide robust classification of images using a relatively small dataset^58^. Likewise, we foresee this approach as being amendable to a wide array of cellular model systems with the caveat that the phenotype is visually apparent and remotely similar to the information learned from the initial training domain^59^. With this potential limitation, it is unknown whether this approach will capture less obvious phenotypes or ones that cannot be characterized visually in advance. One potentially interesting extension to this approach would be to train the convolutional layers of a dNN using images of organoids in order to generate an organoid-specific feature vector. This could potentially further bolster the accuracy of models and provide a route toward building regressive models. However, to achieve this a drastically larger dataset would be needed, which is outside the scope of this study.

The weakly or semi-supervised learning leverages the known experimental structure to generate pseudo-labels for training^60^. Here, the general assumption is that within a given cell model, wells treated under similar conditions will have a similar response pattern, which was visually confirmed for this dataset. Under this logic, one can assign pseudo-labels to the data based on the experimental conditions under which they are run. This type of logic has been successfully deployed in other experimental contexts, which aimed to clustering drugs with similar mechanisms of action^60^. Using those labels, a simple model is constructed with the aim of retrieving images based on content. While this approach has a certain level of imprecision, the implementation of robust models trained across experimental batches with non-overlapping train/testing split can provide a robust estimate of performance.

In this study, we developed a screening approach that combines generic image features from a pre-trained dNN with a k-NN model that was used to map the similarity of experimental treatments to reference controls. We chose to use this approach because the features space produced by extracting information from various levels of dNN architectures has previously demonstrated robust performance in a wide array of image classification tasks as well as equivalent if not better performance when compared to “hand crafted” feature spaces such as scale-invariant features transforms (SIFT), local binary patterns (LBP), and histogram of oriented gradients (HOG)^58,61–63^. The utilization of a k-NN to map unlabeled wells to control-like conditions is further benefitted by providing some level of explanation through the visualization of the nearest neighbors used to determine the classification label while still accounting for visual heterogeneity found across the control conditions^64,65^.

Our collection of *Trp53*-null GEM models displays significant intertumoral heterogeneity which phenocopies human TNBC^45,66^. On examination of tumor-derived 3D organoids, we found that tumor models that are classified as the claudin-low subtype tend to develop a spiky organoid morphology, whereas those that are classified as basal-like tend to form more rounded organoids (Supplementary Fig. 9)^66^. This correlation of organoid spikiness with mesenchymal properties may be due to the high motility and invasiveness of mesenchymal tumor cells in the claudin-low models. Although both T11 and T12 are classified as claudin-low tumors, intertumoral heterogeneity exists which can be implied from the differences in drug response of T11 and T12 cells. For example, the PR50 concentrations for most drugs are lower in T12 than T11 (Fig. 4); BRD inhibitors only inhibited Zeb1 expression in T12 but not T11 cells, and E-cadherin was more readily induced by HDACi in T12 cells as compared to T11 (Fig. 5). These data suggest that different genetic and/or epigenetic landscapes are present in these two phenotypically similar models.

Over the past decades, multiple epigenetic regulators have been identified as modulators of the EMT program in various cancer cell lines in 2D, e.g., HDAC, DNMT, LSD1, KDM6B, PRMT5, EZH2, and G9a^67–76^. However, drug effects are highly context-dependent and mesenchymal cells near the end of EMT spectrum are especially difficult to be reprogrammed. The fact that none of the HMT and KDM inhibitors showed up as positive hits in these screens implied that HMT and KDM may be less important than class I HDAC and BRD proteins in maintaining the EMT status of claudin-low cells, and that targeting HMT or KDM may provide less benefit in improving the chemotherapeutic response in vivo. As a discovery platform to identify new therapeutics, additional libraries can be analyzed using this platform besides the small focused epigenetic drug library employed in this study. This approach may also be applied to FDA-approved drugs to facilitate rapid drug repurposing.

## Methods

### Establishment of primary *Trp53*-null mammary tumor cell lines

The *Trp53*-null GEM models were previously established by transplantation of *Trp53*-deleted donor mammary epithelium into syngeneic BALB/c hosts, which gave rise to a bank of heterogenous mammary tumors^41,42^. The genetic engineering event that led to the deletion of *Trp53* alleles brought in a neomycin resistance gene, which facilitated the isolation of tumor cell lines by G418 selection. Primary tumor cells were isolated by digesting tumor tissues in 1 mg/ml Collagenase A (#11088793001, Sigma-Aldrich) for 2 h at 37 °C with 125 rpm rotation and cultured in medium containing 50 μg/ml of G418 (#10131035, Thermo Fisher) for 2 weeks. All established cell lines were tested free of mycoplasma contaminants using the Universal Mycoplasma detection kit (#30-1012 K, ATCC).

### Cell and organoid culture

All primary cells were cultured in DMEM/F-12 medium (#11330032, Thermo Fisher) supplemented with 10% fetal bovine serum (FBS, #F0900-050, GenDEPOT), 5 µg/ml insulin (#I-5500, Sigma), 1 µg/ml hydrocortisone (#H0888, Sigma), 10 ng/ml epidermal growth factor (EGF, #SRP3196, Sigma), and 1X Antibiotic-Antimycotic (#15240062, Thermo Fisher). The lentiviral plasmid pINDUCER13 miR-200c-141 (#81020, Addgene) was used to generate T11 and T12-TetOn-miR-200c inducible cell lines. The Z-cad reporter plasmids FUGW-d2GFP-ZEB1 3′UTR (#79601, Addgene) and pHAGE-E-cadherin-RFP (#79603, Addgene) were used to generate the T11-TetOn-miR-200c-Z-cad cell line. For organoid culture, cell culture plates were first coated with 5 µl of Growth Factor Reduced Matrigel (#354230, Corning) to avoid cell settlement to the bottom of the plate. Next, the coating was allowed to solidify at 37 °C for 15 min. Then a 40 µl droplet of Matrigel containing 1000 trypsinized tumor cells was plated and solidified at 37 °C for 30 min before the culture medium was added. Organoid formation was monitored daily with medium changes every 2–3 days. For miR-200 induction or drug treatment, doxycycline (1 µg/ml) or drugs were added to culture medium 2 days after cell seeding. For qPCR and immunoblotting assays, organoids were retrieved with Cell Recovery Solution (#354253, Corning) and washed with PBS prior to lysis. For analyzing *CDH1* promoter-driven RFP reporter activities, cells were treated with drugs for 48 h and subjected to flow cytometry. Flow data were analyzed with FlowJo software (version 10.6).

### Immunofluorescence (IF) and immunohistochemistry (IHC)

Organoids were fixed in fresh 4% paraformaldehyde (PFA) for 10 min and embedded in HistoGel (#HG-4000-012, Thermo Fisher) before paraffinization and embedding. Tumor tissue specimens were fixed in 4% PFA for 24 h and stored in 70% ethanol until paraffin embedding. Organoid and tissue sections were deparaffinized, rehydrated, and subjected to antigen retrieval in Tris-EDTA (pH 9.0) buffer for 20 min in a steamer. Slides were incubated with primary antibodies overnight at 4 °C and secondary antibodies for 1 h at room temperature. Antibodies and concentrations were: Keratin 8 (IF 1:250, #TROMA-1, Developmental Studies Hybridoma Bank), E-cadherin (IF 1:200, #610182, BD Biosciences), and pan cytokeratin (IHC, 1:500, #ab9377, Abcam).

### F-actin staining of whole-mount organoids

Organoids cultured on chamber slides (#C7182, Thermo Fisher) were fixed in 4% PFA for 15 min and permeabilized in 0.1% Triton X-100 in PBS for 15 min. Then samples were incubated with Alexa Fluor 488 Phalloidin (#A12379, Thermo Fisher) for 30 min at room temperature and counterstained with DAPI (#R37606, Thermo Fisher). Images were taken using a confocal laser scanning microscope with 20X objective (Nikon A1-Rs) and movies were generated through Z-stack imaging.

### Immunoblotting assay

Tumors that reached ethical endpoint were snap-frozen upon harvest and homogenized in lysis buffer (Tris-HCl pH 6.8, 62.5 mM; SDS, 2%) using zirconium beads (#D1132-30, Benchmark Scientific) and a bead homogenizer. Protein concentrations were measured with BCA Protein Assay Kit (#23227, Thermo Fisher). Whole-cell extracts were separated by SDS-polyacrylamide gels and transferred to polyvinylidene difluoride membranes (#IPVH00010, Millipore). Antibodies and dilutions were E-cadherin (1:1000, #3195, Cell Signaling Technology (CST)), Keratin 8 (1:1000, #TROMA-1, Developmental Studies Hybridoma Bank), Zeb1 (1:1000, #3396, CST), GAPDH (1:3000, #2118, CST), Slug (1:1000, #9585, CST), AcH3 (1:2000, #06-599, Upstate), and AcH4 (1:2000, #06-598, Upstate). Uncropped scans of all the blots are provided in the Source data file.

### Quantitative real-time PCR (qPCR)

Total RNA was extracted using TRIzol reagent (#15596026, Thermo Fisher) following the manufacturer’s instructions. Reverse transcription for miRNAs was carried out using the Taqman microRNA Reverse Transcription kit (#4366596, Thermo Fisher) with corresponding TaqMan Small RNA Assays. TaqMan Universal Master Mix (#4440040, Thermo Fisher Scientific) was utilized for miRNA qPCR reactions. The TaqMan RT primers and qPCR probes and primers used in this study were U6 snRNA (assay ID 001973, Thermo Fisher) and miR-200c (assay ID 002300, Thermo Fisher). For qPCR analysis of mRNA, 1 µg of total RNA was converted to cDNA using the High-Capacity cDNA Reverse Transcription Kit (#4368814, Thermo Fisher). mRNA levels were detected using amfisure qGreen Q-PCR Master Mix (#Q5602, GenDEPOT). *Actb* was used as an internal reference. The levels of target genes were normalized to the levels of internal reference gene to permit the calculation of the 2^−ΔΔCt^ value. The sequences of all primers are listed in Supplementary Data 3.

### Preparation of 384-well organoid plates

For the primary screening and secondary validation assays, Matrigel (with 9–10 mg/ml protein concentration) was first diluted with culture medium to result in a final dilution factor of 60% (v/v) for coating purpose. Next, 10 µl of the 60% Matrigel (with 5–6 mg/ml protein concentration) was added to 384-well uClear plates (#781091, Greiner) using a Multidrop liquid dispenser (Thermo) and was allowed to solidify at 37 °C for at least 30 min. Single-cell suspensions were then counted using the TC20 (Bio-Rad) automated cell counter with Trypan Blue staining as described in the manufacturer’s instructions. The single-cell suspensions were then diluted with media to a concentration of 8.5 × 10^4^ cells/ml and further mixed with Matrigel to a final dilution of 50% (v/v, with 4–4.5 mg/ml Matrigel protein concentration). A total of 12 µl of cell-containing Matrigel was overlaid into the pre-coated 384-well plates using a chilled Multidrop liquid dispenser, resulting in a seeding density of 500 cells/well. Seeded plates were then incubated at 37 °C for 30 min, after which 80 μl of cell culture medium was added to the top of the well. After allowing cells to recover for two days, plates were treated with pre-arrayed drug libraries using a pin-transfer (Tecan).

### Drug addition for screening and validation assays

A pre-arrayed epigenetic drug library was provided by the Institute for Applied Cancer Science (IACS) at MD Anderson Cancer Center. All drugs were suspended in 100% DMSO to a concentration of 10 mM in 96-well source plate. For dose–response curves, serial dilutions were made in DMSO in half-log steps (3.3X) into separate 96-well plates. Then 100 nl of material was transferred from the drug plates into the assay plates using a Pin tool (Tecan). Measurements were performed in technical quadruplicate in 10 doses and replicate AUC values were highly correlated. For doxycycline control wells, 10 mg/ml stock solution was diluted 100X and 10 µl was added into the well to reach a final 10 µg/ml concentration. At the end of the imaging assay, a CellTiter-Glo assay was performed according to the manufacture’s instructions. Briefly, CellTiter-Glo 3D (#G9682, Promega) was overlaid into wells using a 1:1 (v/v) with media. The plates were then incubated at room temperature for 30 min, after which luminescence was read using a Tecan Infinite M1000 plate reader.

### Image acquisition and analysis

A z-stack of 18 bright-field images sampled at 100 µm interval were acquired using 4X Nikon Plan APO (NA = 0.2) objective on ImageXpress Microconfocal (Molecular Devices) with software MetaXpress (version 6.5.4.532) to ensure that the full volume of Matrigel was acquired. Images were pre-processed using an ImageJ (version 1.48e) macro^77,78^. In brief, the image stack was first inverted. Next, a rolling ball background subtraction was performed using a radius of 10 pixels. The stack focuser plugin^79^ was then used to project the corrected z-stack images into a single z-plane. To scale up the analysis, the ImageJ macro was parallelized using Pipeline Pilot 2018 golden server edition (Dassault Systems). Projected images were then read into python 3.8.5 environment and converted into RGB images using numpy (version 1.9.2) to fit the requirements used by the RESNET architecture. The pre-weighted RESNET-18 was downloaded using PyTorch 1.7.1 interface and image embedding generated using methods adapted from https://github.com/christiansafka/img2vec. This resulted in a 512-dimensional vector that was saved into a CSV file and subsequently read back into Pipeline Pilot where it was merged with other plate metadata (positions of control/drug-treated wells, concentrations, etc). The data were then randomly split in half for each control category and a k-NN model was trained using the 6 nearest neighbors with the knn3 function in the *caret* (version 6.0-86) package^80^ built for R (version 3.6.2). Series of models were trained from data within and across assay batches to evaluate the potential influence of batch effects. For models trained at the batch level, 8 images per control were used for training and 8 images per control were used for testing. The only exception to this was in the T11 Run2 where the media control was excluded because that region of the plate was seeded with cells. For cumulative models, 16 images per control were used for training and 16 images per control were used for testing. For all models, the performance was determined by calculating the accuracy of the trained model on the withheld test set which are the values that are reported.

### Tumor transplantation and treatment

The animal study was performed in compliance with the rules of the Guide for the Care and Use of Laboratory Animals of the NIH. All mice were maintained and sacrificed according to guidelines of IACUC at Baylor College of Medicine (Protocol AN-504). Mice were maintained in a room with a 14-h light/10-h dark cycle and a temperature of 68–72 °F with 30–70% humidity. T12 tumors were dissociated into single tumor cells using collagenase as reported previously^66^. Tumor cells (25,000) were transplanted to the mammary fat pad of 6–8-week-old female BALB/c mice (Envigo). After 9 days of transplantation, tumors became palpable with an average size of 50 mm^3^ using a calculation of ½ length × width^2^. Treatment then was initiated with the following drugs and dosages: Azacytidine (#A2385, Sigma, 0.5 mg/kg), Mocetinostat (#M2433, LC Laboratories, 5 mg/kg), and Carboplatin (#C2538, Sigma, 50 mg/kg dosed weekly). All three drugs were delivered via intraperitoneal injection. The ethical endpoint was reached when tumors reached a 1.5-cm diameter.

### Statistical analysis

Q-PCR data were presented as the mean ± standard error of the mean (s.e.m.). Unpaired two-tailed Student’s *t*-tests were performed to compare the differences between two groups. For the AUC values calculated in Fig. 3, support vector regression was used to provide a robust curve fit of the probability scores produced by the k-NN model across the tested concentrations. To achieve this, a two-fold cross-validated SVM regression model with a radial kernel was trained on data using the formula “Class probability ~ Concentration” of the *svm* function found in the R package *e1071* (version 1.7-6)^81^. This effectively results in generation of a non-linear hyperplane that is analogous to a fitted dose–response curve^50^. The curves presented in Fig. 4 are constructed using a constrained 4-parameter logistic model where the top and bottom are limited to be between 0 and 1 using Prism (GraphPad, version 8.4.3). The log-rank test was used to test for the significant differences of Kaplan–Meier survival curves between groups.

### Reporting summary

Further information on research design is available in the Nature Research Reporting Summary linked to this article.

## Supplementary information

Supplementary Information

Description of Additional Supplementary Files

Supplementary Data 1

Supplementary Data 2

Supplementary Data 3

Supplementary Movie 1.

Supplementary Movie 2.

Supplementary Movie 3.

Supplementary Movie 4.

Supplementary Movie 5.

Reporting Summary

## Data Availability

All the data generated in this study are available within the article and its supplementary information and from the corresponding author upon reasonable request. A reporting summary for this article is available as a Supplementary Information file. Source data are provided with this paper.

## Source data

Source Data

## Code availability

Code, raw, and processed data are available at https://github.com/ReidTPowell/Organoid-analysis. Workflows generated with Pipeline Pilot will require access to a Pipeline Pilot 2018 server and professional client.
