# Supplementary material for: Morphological screening of mesenchymal mammary tumor organoids to identify drugs that reverse epithelial-mesenchymal transition
Source: Nat Commun. 2021 Jul 12;12:4262. doi: 10.1038/s41467-021-24545-3 (PMC8275587; doi:10.1038/s41467-021-24545-3)
Supplement: Supplementary file 1 — Supplementary Information [file 41467_2021_24545_MOESM1_ESM.pdf]

Supplementary Information

Morphological screening of mesenchymal mammary tumor organoids to identify drugs that reverse epithelial-mesenchymal transition

Na Zhao[1#], Reid T. Powell[2#], Xueying Yuan[1], Goeun Bae[2], Kevin P. Roarty[1], Fabio Stossi[1,3], Martina Strempfl[4], Michael J. Toneff[5], Hannah L. Johnson[3], Sendurai A. Mani[6], Philip Jones[7], Clifford C. Stephan[2], Jeffrey M. Rosen[1*]

1. Department of Molecular and Cellular Biology, Baylor College of Medicine, Houston, Texas, USA.

2. Center for Translational Cancer Research, Texas A&M Health Science Center, Institute of Biosciences and Technology, Houston, Texas, USA.

3. Integrated Microscopy Core, Baylor College of Medicine, Houston, Texas, USA.

4. Graz University of Technology, NAWI Graz, Graz, Styria, Austria.

5. Widener University, Chester, Pennsylvania, USA.

6. Department of Translational Molecular Pathology, University of Texas MD Anderson Cancer Center, Houston, Texas, USA.

7. Institute of Applied Cancer Science (IACS), University of Texas MD Anderson Cancer Center, Houston, Texas, USA.

[#] These authors contributed equally to this work.

* Correspondence to Jeffrey M. Rosen jrosen@bcm.edu

Supplementary Fig. 1: miR-200c expression changes mesenchymal organoid morphology.

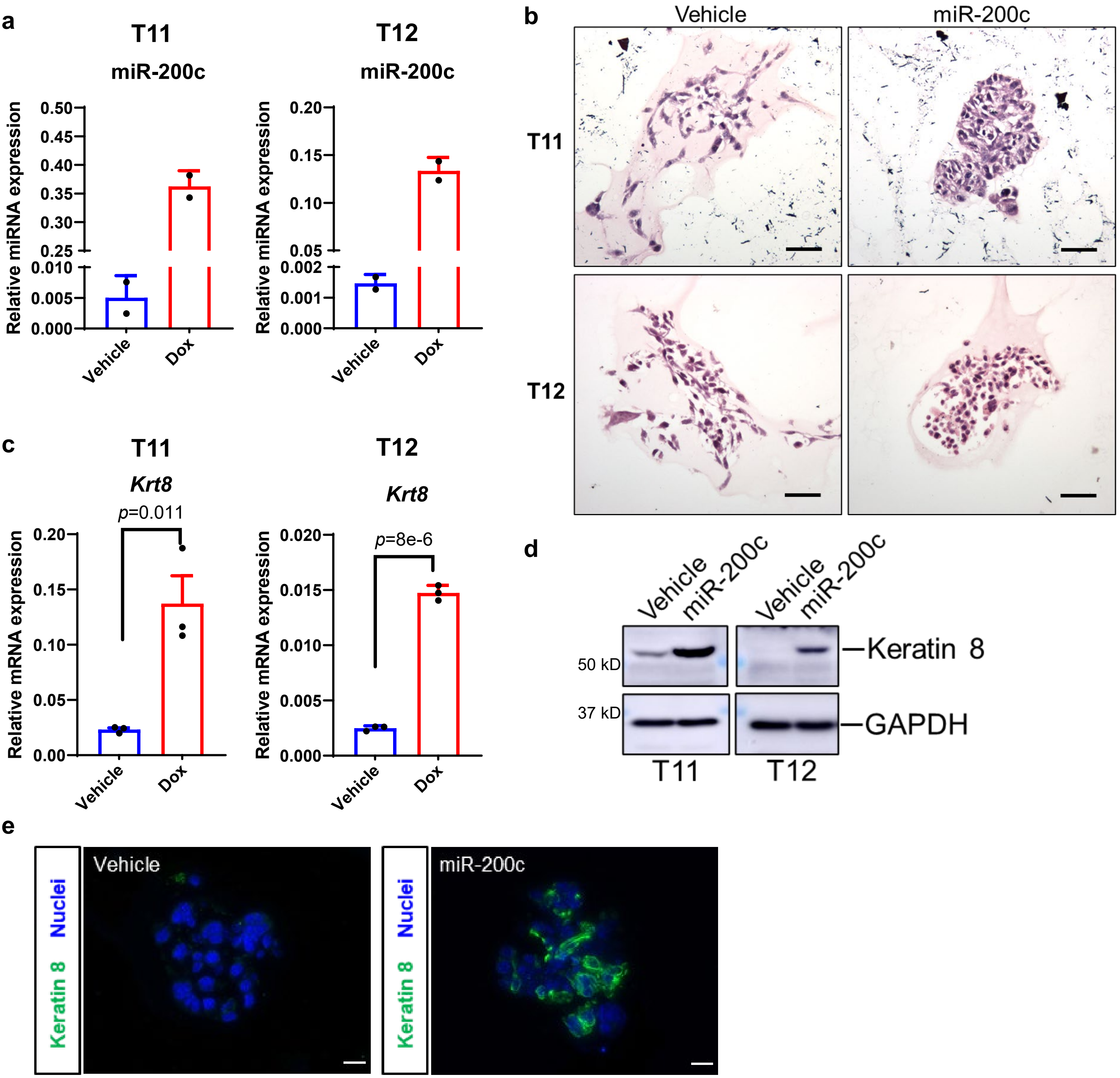

Supplementary Fig. 1: miR-200c expression changes mesenchymal organoid morphology.

a. qPCR of miR-200c levels in vehicle- or dox-treated T11 and T12 cells. Data are presented relative to *U6* and shown as mean ± s.e.m. of independent biological duplicates.

b. H&E staining of vehicle- or dox-treated T11 and T12 organoid sections. Representative images of independent biological triplicates. Scale bar: 50 μm.

c. qPCR of *Krt8* levels in vehicle- or dox-treated T11 and T12 cells. Data are presented relative to *Gapdh* and shown as mean ± s.e.m. of independent biological triplicates. Statistical analysis by unpaired Student t-tests (two-tailed).

d. Immunoblotting assay of Keratin 8 in vehicle- or dox- treated T11 and T12 cells. Representative images of independent biological duplicates.

e. IF staining of Keratin 8 in vehicle- or dox-treated T12 organoid sections. Representative images of independent biological triplicates. Scale bar: 20 μm.

Supplementary Fig. 2: miR-200c induced F-actin reorganization and E-cadherin expression.

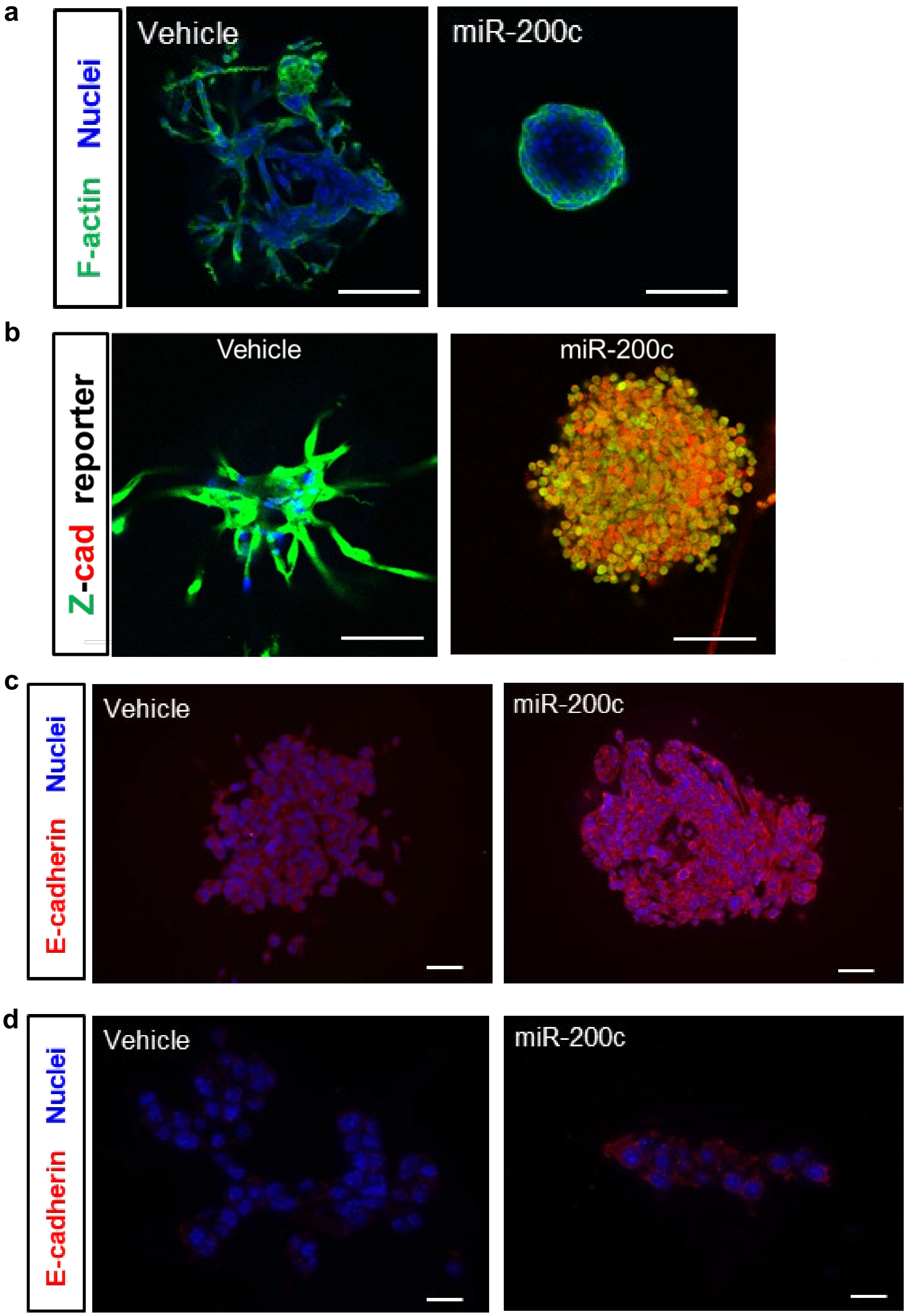

Supplementary Fig. 2: miR-200c induced F-actin reorganization and E-cadherin expression.

a. F-actin staining of vehicle- or dox-treated T12 whole-mount organoids. Representative images of independent biological triplicates. Scale bar: 100 μm.

b. Z-cad reporter imaging in vehicle- or dox-treated T12 whole-mount organoids. Representative images of independent biological triplicates. Scale bar: 100 μm.

c. IF staining of E-cadherin in vehicle- or dox-treated T11 organoid sections. Representative images of independent biological triplicates. Scale bar: 50 μm.

d. IF staining of E-cadherin in vehicle- or dox-treated T12 organoid sections. Representative images of independent biological triplicates. Scale bar: 20 μm.

Supplementary Fig. 3: Adaptation to High-throughput 3D organoid culture.

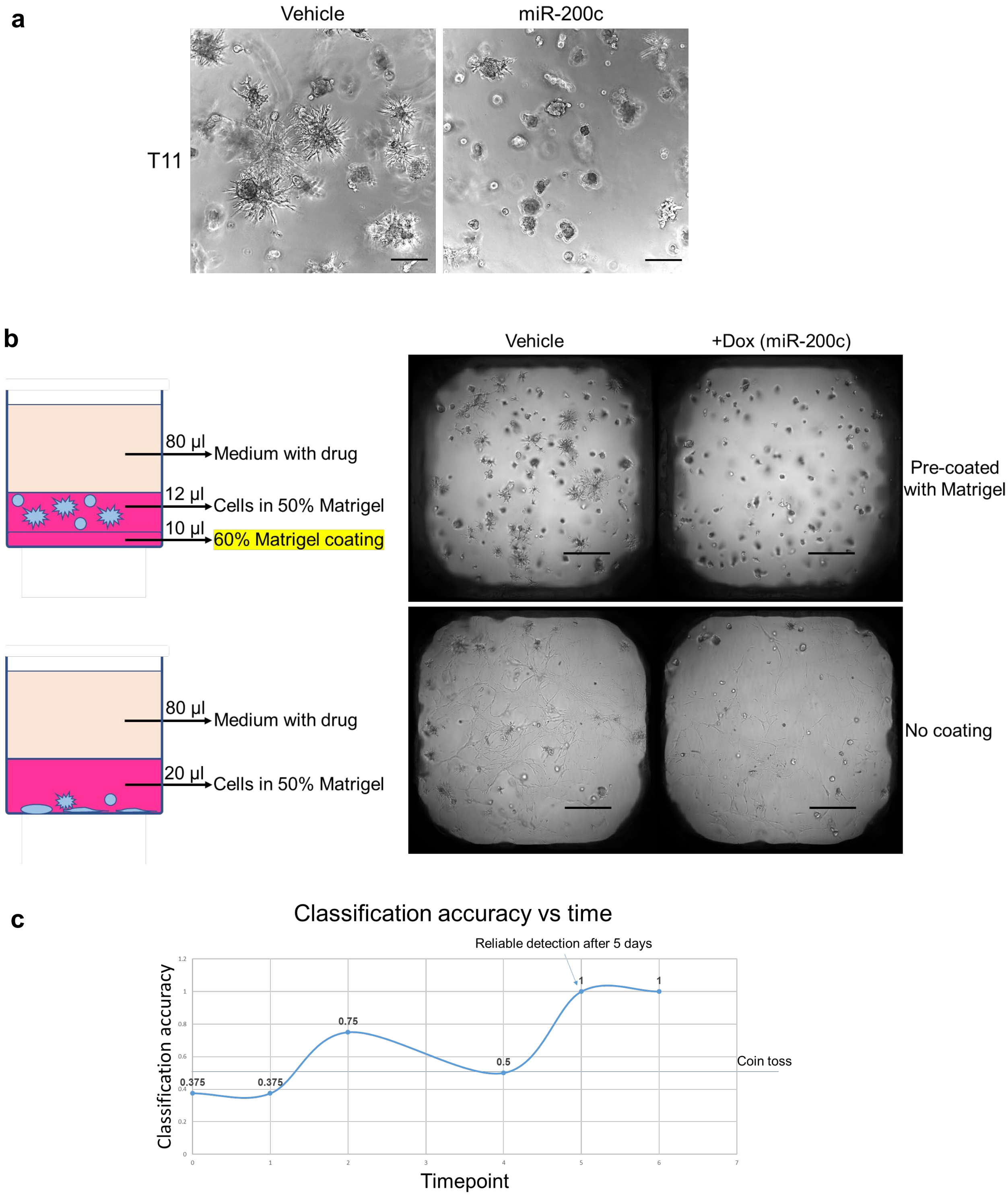

Supplementary Fig. 3: Adaptation to High-throughput 3D organoid culture.

a. Morphology of vehicle- or dox- treated T11 organoids in 50% Matrigel by phase contrast microscopy. Representative images of independent biological triplicates. Scale bar: 200 µm.

b. Left, schematic diagram of organoid cultures in 384 well plate with or without 60% Matrigel coating. Right, representative images of control wells. Representative images of technical replicates (n=8). Scale bar: 200 µm.

c. Classification accuracy of CNN model over time. Data shows controls are fully distinguishable after 5 days.

Supplementary Fig. 4: Modeling statistics for the screen

ResNET-kNN performance for T11 test set

1st Run

Confusion matrix				Balanced Accuracy	Balanced Error Rate	Informedness
Act/Pred	media	neg	pos	1.00	0.00	1.00
media	8	0	0			
neg	0	8	0			
pos	0	0	8			

2nd Run

Confusion matrix				Balanced Accuracy	Balanced Error Rate	Informedness
Act/Pred	media	neg	pos	1.00	0.00	1.00
media	0	0	0			
neg	0	8	0			
pos	0	0	8			

Both

Confusion matrix				Balanced Accuracy	Balanced Error Rate	Informedness
Act/Pred	media	neg	pos	0.90	0.10	0.79
media	8	0	0			
neg	0	15	1			
pos	0	4	12			

ResNET-kNN performance for T12 test set

1st Run

Confusion matrix				Balanced Accuracy	Balanced Error Rate	Informedness
Act/Pred	media	neg	pos	1.00	0.00	1.00
media	8	0	0			
neg	0	8	0			
pos	0	0	8			

2nd Run

Confusion matrix				Balanced Accuracy	Balanced Error Rate	Informedness
Act/Pred	media	neg	pos	0.83	0.17	0.67
media	8	0	0			
neg	1	6	1			
pos	2	0	6			

Both

Confusion matrix				Balanced Accuracy	Balanced Error Rate	Informedness
Act/Pred	media	neg	pos	0.92	0.08	0.83
media	16	0	0			
neg	2	14	0			
pos	2	0	14			

Iterative validation

T11

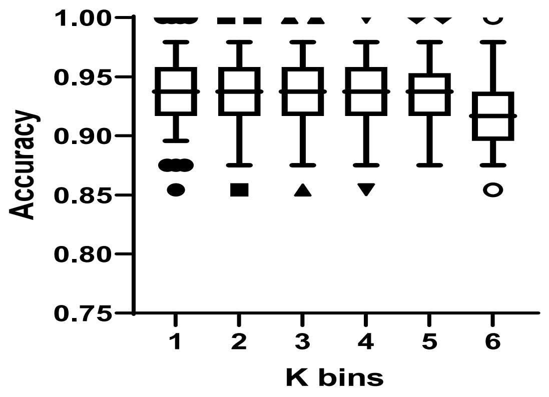

T12

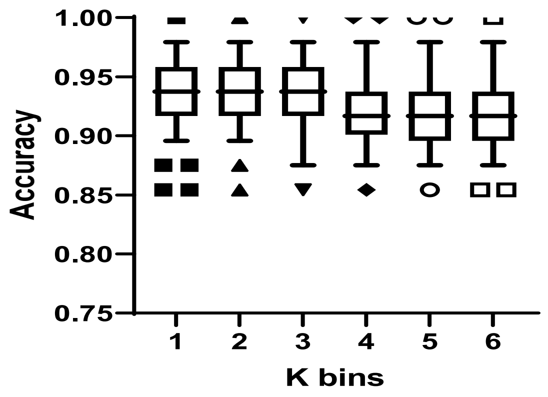

Validation

T11:

Confusion matrix				Balanced Accuracy	Balanced Error Rate	Informedness
Act/Pred	media	neg	pos	1.00	0.00	1.00
media	8	0	0			
neg	0	8	0			
pos	0	0	8			

T12:

Confusion matrix				Balanced Accuracy	Balanced Error Rate	Informedness
Act/Pred	media	neg	pos	1.00	0.00	1.00
media	8	0	0			
neg	0	8	0			
pos	0	0	8			

Supplementary Fig. 4: RESNET-kNN model evaluation

Biological replicates are obtained from two different experimental batches for the T11 and T12 cells. Accordingly, a series of models are trained using half of the controls from within (denoted as 1st and 2nd run) and across biological batches (denoted as "Both") for each cell model. The media control for the 2nd run of T11 was excluded because wells were seeded with cells. To further validate this method, 100 K-NN models are trained in the "both" dataset that was randomly sub-sampled while also varying the K parameter from 1 to 6. Finally, a separate model was then used for the validation assay to show independent replication of the primary screen. All the data shown above are from the application of the trained model on the with-held test set. The whiskers are the 10th and 20th percentiles, and the boxes are the 25th, 50th, and 75th percentiles.

Supplementary Fig. 5: Dose response curves for cell toxicity and positive-like phenotype.

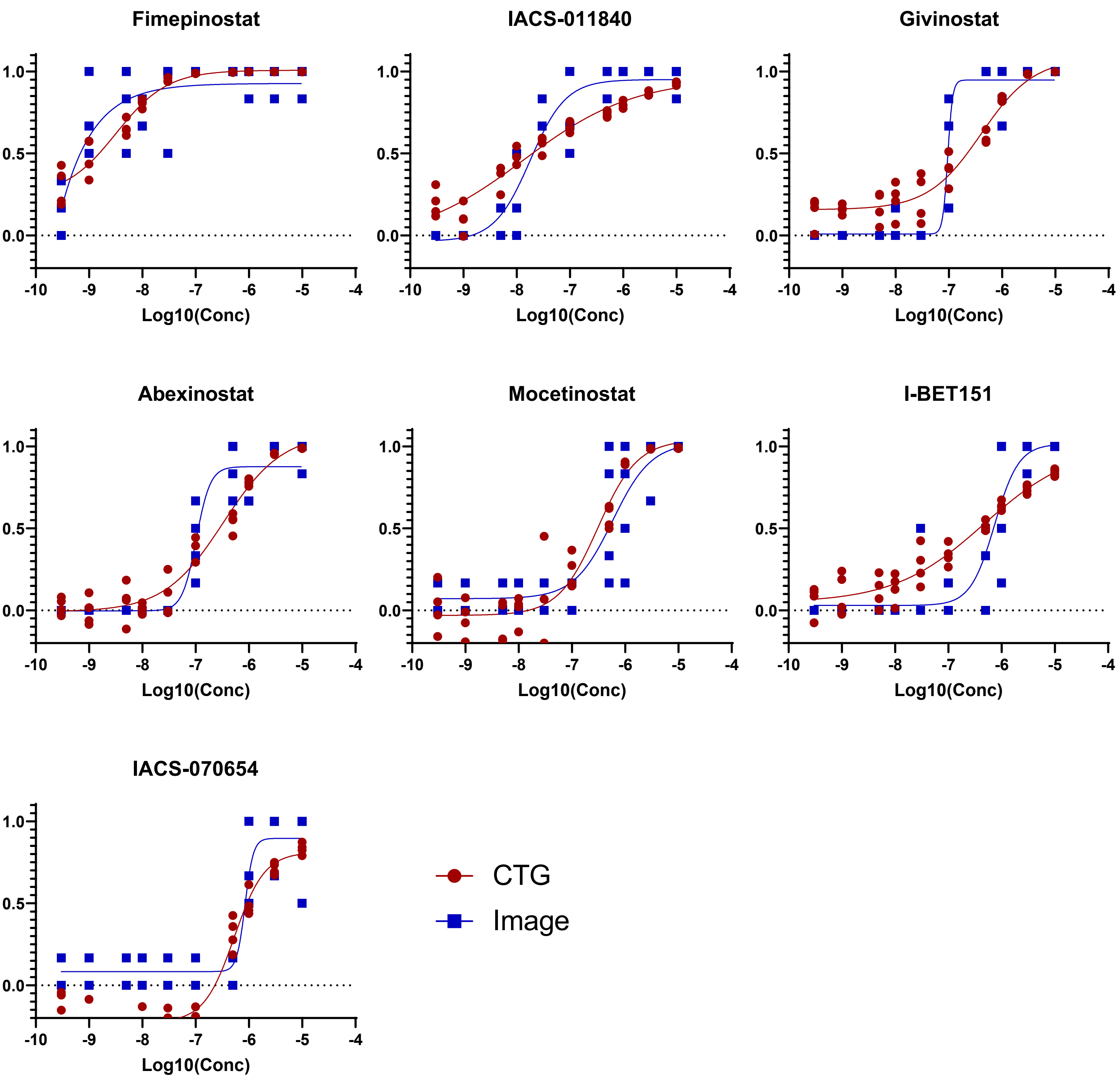

Supplementary Fig. 5: Dose response curves for cell toxicity and positive-like phenotype.

Dose response curves of drug toxicity indicated by Cell TiterGlo measurements (CTG, red line) and the probability resembling the positive control (Image, blue line) in T11 organoids.

Supplementary Fig. 6: Effects of top drug hits on *Cdh1* expression.

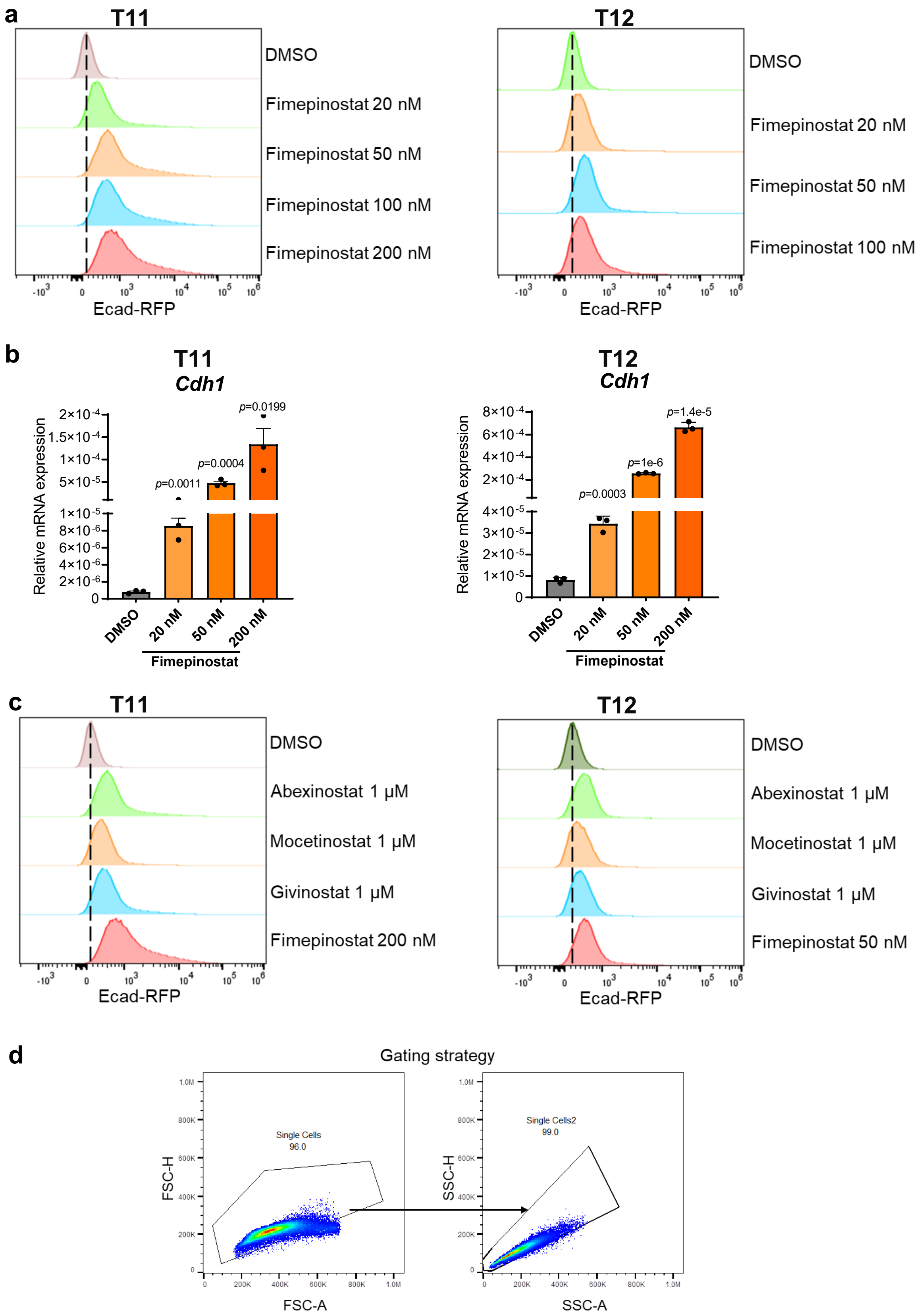

Supplementary Fig. 6: Effects of top drug hits on *Cdh1* expression.

a. Flow cytometry of *CDH1* promoter-driven RFP reporter activity in Fimepinostat-treated T11 and T12 cells.

b. qPCR of *Cdh1* in Fimepinostat-treated T11 and T12 cells. Data are presented relative to *Actb* and shown as mean ± s.e.m. of independent biological triplicates. Statistical analysis by unpaired Student *t*-tests (two-tailed).

c. Flow cytometry of *CDH1* promoter-driven RFP reporter activity in HDAC inhibitor-treated T11 and T12 cells.

d. Gating strategy of flow cytometry in a and c. Single cells were gated and analyzed.

Supplementary Fig. 7: The effects of Azacytidine on T12.

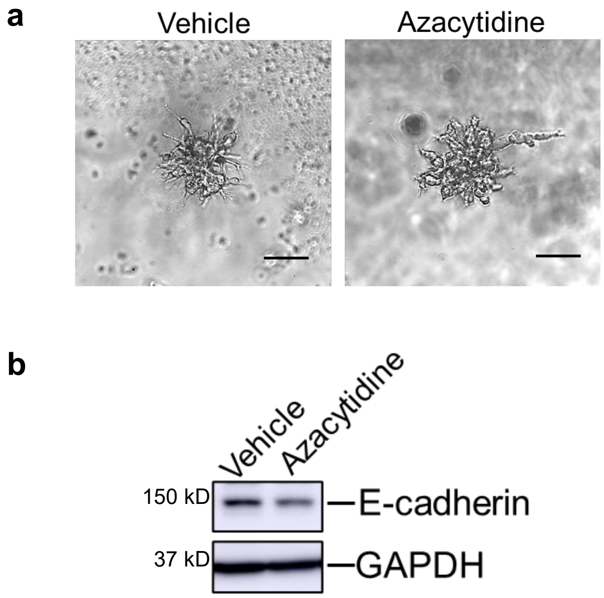

Supplementary Fig. 7: The effects of Azacytidine on T12.

a. Morphology of vehicle- or Azacytidine (0.5 μM)- treated T12 organoids in 100% Matrigel by phase contrast microscopy. Representative images of independent biological triplicates. Scale bar: 50 μm.

b. Immunoblotting assay of E-cadherin in T12 cells treated with vehicle or Azacytidine (0.5 μM) for 48 hours. Representative images of independent biological triplicates.

Supplementary Fig. 8: Epigenetic therapies reversed EMT in T12 tumors.

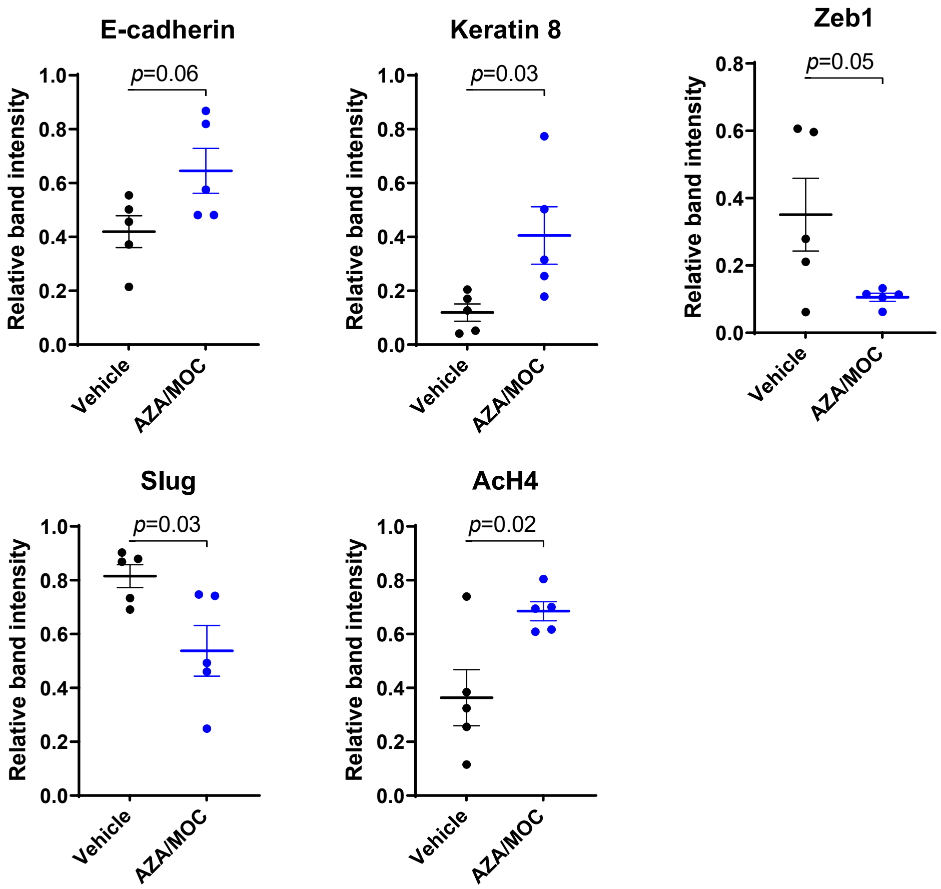

Supplementary Fig. 8: Epigenetic therapies reversed EMT in T12 tumors.

Quantification of immunoblotting assays in Fig. 6b. Band intensities were quantified using Image Lab and normalized to GAPDH. *P* value was calculated using unpaired Student *t*-tests (two-tailed). *n*=5 for each group.

Supplementary Fig. 9: Characterization of *Trp53*-null mammary tumors and organoids.

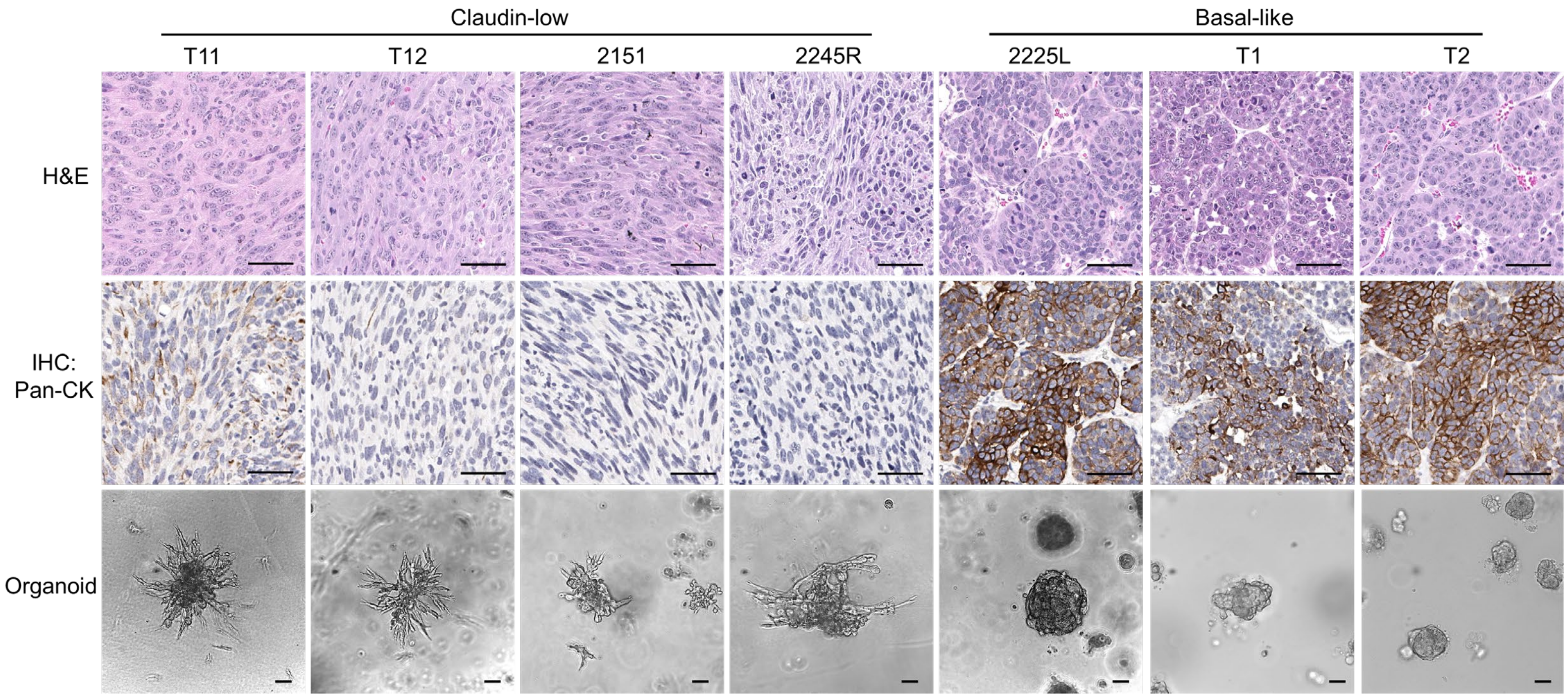

Supplementary Fig. 9: Characterization of *Trp53*-null mammary tumors and organoids.

Characterization of *Trp53*-null GEM tumors and organoids. Upper and middle panels: H&E staining and IHC staining of pan-cytokeratin in claudin-low and Basal-like *Trp53*-null GEM tumors. Representative images of independent biological duplicates. Lower panel: morphology of tumor-derived organoids. Representative images of independent biological triplicates. Scale bar: 50 µm.
