# Supplementary material for: Morphological screening of mesenchymal mammary tumor organoids to identify drugs that reverse epithelial-mesenchymal transition
Source: Nat Commun. 2021 Jul 12;12:4262. doi: 10.1038/s41467-021-24545-3 (PMC8275587; doi:10.1038/s41467-021-24545-3)
Supplement: Supplementary file 2 — Description of Additional Supplementary Files [file 41467_2021_24545_MOESM2_ESM.pdf]

Description of Additional Supplementary Files

File Name: Supplementary Data 1
Description: Information on the epigenetic drug library.

File Name: Supplementary Data 2
Description: Drug response profiles.

File Name: Supplementary Data 3
Description: Primers used in qPCR.

File Name: Supplementary Movie 1
Description: Longitudinal comparison of T11 organoids.

File Name: Supplementary Movie 2
Description: Z-stack reconstruction of vehicle-treated T11 organoids with F-actin staining.

File Name: Supplementary Movie 3
Description: Z-stack reconstruction of miR-200c-induced T11 organoids with F-actin staining.

File Name: Supplementary Movie 4
Description: Z-stack reconstruction of vehicle-treated T12 organoids with F-actin staining.

File Name: Supplementary Movie 5
Description: Z-stack reconstruction of miR-200c-induced T12 organoids with F-actin staining.
