# Supplementary figures and images for: Morphological screening of mesenchymal mammary tumor organoids to identify drugs that reverse epithelial-mesenchymal transition
Source: Nat Commun. 2021 Jul 12;12:4262. doi: 10.1038/s41467-021-24545-3 (PMC8275587; doi:10.1038/s41467-021-24545-3)

## Slide 1
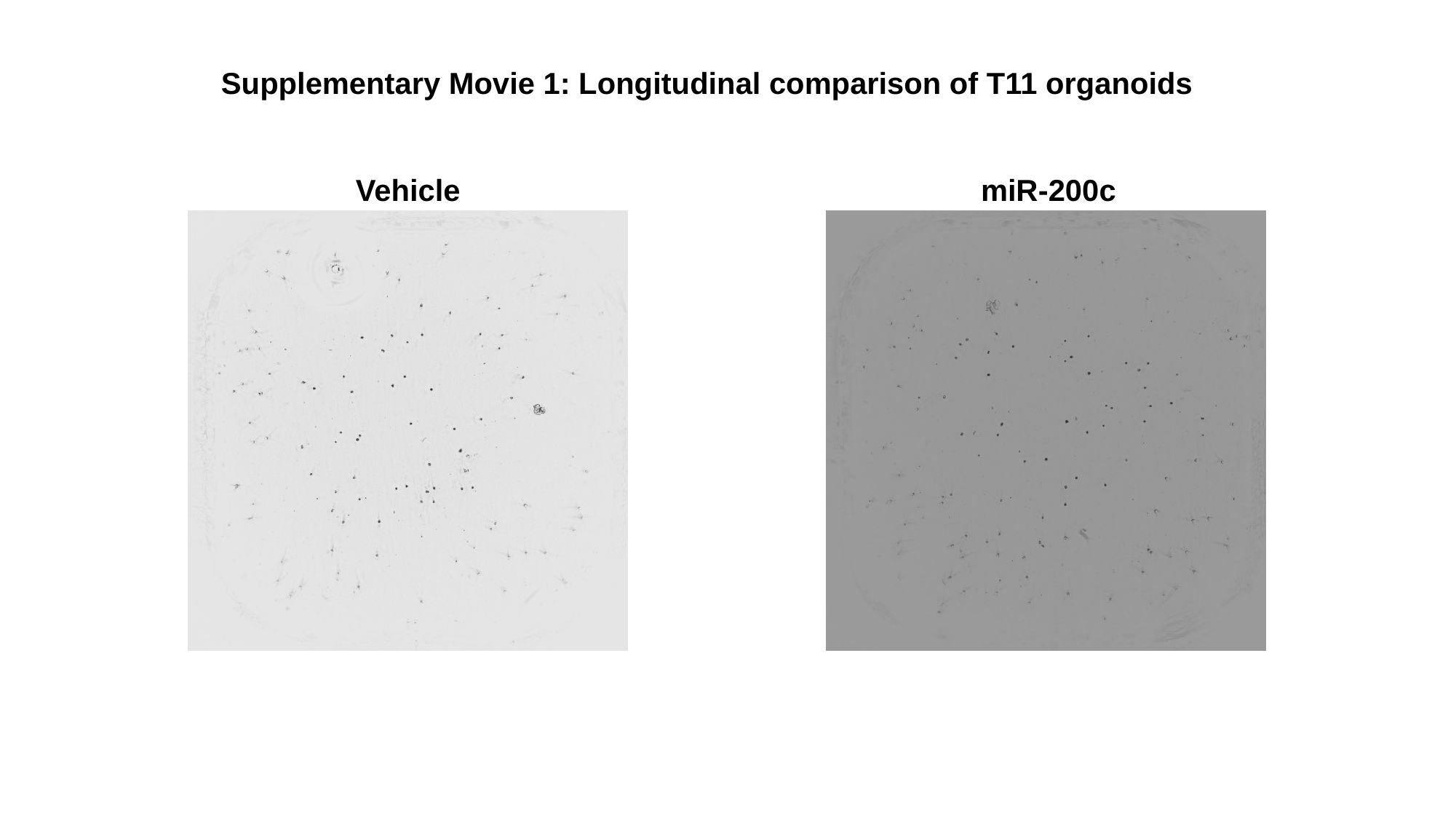

Supplementary Movie 1: Longitudinal comparison of T11 organoids
miR-200c
Vehicle

Supplement: Supplementary file 6 — Supplementary Movie 1. [file 41467_2021_24545_MOESM6_ESM.pptx]
