# Supplementary material for: Morphological screening of mesenchymal mammary tumor organoids to identify drugs that reverse epithelial-mesenchymal transition
Source: Nat Commun. 2021 Jul 12;12:4262. doi: 10.1038/s41467-021-24545-3 (PMC8275587; doi:10.1038/s41467-021-24545-3)
Supplement: Supplementary file 11 — Reporting Summary [file 41467_2021_24545_MOESM11_ESM.pdf]

nature research

Corresponding author(s): Jeffrey M. Rosen

Last updated by author(s): Jun 9, 2021

Reporting Summary

Nature Research wishes to improve the reproducibility of the work that we publish. This form provides structure for consistency and transparency in reporting. For further information on Nature Research policies, see Authors & Referees and the Editorial Policy Checklist .

Statistics

For all statistical analyses, confirm that the following items are present in the figure legend, table legend, main text, or Methods section.

n/a	Confirmed	
☐	☒	The exact sample size (*n*) for each experimental group/condition, given as a discrete number and unit of measurement
☐	☒	A statement on whether measurements were taken from distinct samples or whether the same sample was measured repeatedly
☐	☒	The statistical test(s) used AND whether they are one- or two-sided<br>*Only common tests should be described solely by name; describe more complex techniques in the Methods section.*
☐	☒	A description of all covariates tested
☐	☒	A description of any assumptions or corrections, such as tests of normality and adjustment for multiple comparisons
☐	☒	A full description of the statistical parameters including central tendency (e.g. means) or other basic estimates (e.g. regression coefficient) AND variation (e.g. standard deviation) or associated estimates of uncertainty (e.g. confidence intervals)
☐	☒	For null hypothesis testing, the test statistic (e.g. *F*, *t*, *r*) with confidence intervals, effect sizes, degrees of freedom and *P* value noted<br>*Give P values as exact values whenever suitable.*
☒	☐	For Bayesian analysis, information on the choice of priors and Markov chain Monte Carlo settings
☐	☒	For hierarchical and complex designs, identification of the appropriate level for tests and full reporting of outcomes
☒	☐	Estimates of effect sizes (e.g. Cohen's *d*, Pearson's *r*), indicating how they were calculated

Our web collection on statistics for biologists contains articles on many of the points above.

Software and code

Policy information about availability of computer code

Data collection	HTS imaging data was collected using an imageXpress microconfocal using MetaXpress (6.5)
Data analysis	Data analysis was performed using the following open source software: Python (3.8.2), PyTorch (1.7.1), Numpy (1.9.2), R (3.6.2), caret (6.0-86), e1071 (1.7-6), and ImageJ (1.48e). Commercial software include Graphpad Prism 9 and Biovia Pipeline Pilot 2018 golden server edition. Flow cytometry data was analyzed using FlowJo (10.6). Custom scripts developed in Python, R and Pipeline Pilot with accompanying example data are available at: https://github.com/ReidTPowell/Organoid-analysis

For manuscripts utilizing custom algorithms or software that are central to the research but not yet described in published literature, software must be made available to editors/reviewers. We strongly encourage code deposition in a community repository (e.g. GitHub). See the Nature Research guidelines for submitting code & software for further information.

Data

Policy information about availability of data

All manuscripts must include a data availability statement. This statement should provide the following information, where applicable:

- Accession codes, unique identifiers, or web links for publicly available datasets
- A list of figures that have associated raw data
- A description of any restrictions on data availability

The data that support the findings of this study are included with the manuscript and are also available for download at: https://github.com/ReidTPowell/Organoid-analysis

Field-specific reporting

Please select the one below that is the best fit for your research. If you are not sure, read the appropriate sections before making your selection.

- [x] Life sciences
- [ ] Behavioural & social sciences
- [ ] Ecological, evolutionary & environmental sciences

For a reference copy of the document with all sections, see nature.com/documents/nr-reporting-summary-flat.pdf

Life sciences study design

All studies must disclose on these points even when the disclosure is negative.

Sample size	In general, no calculations were done to determine sample size. Sample size was determined based on standards for experimental cancer cell biology and animal studies, attempting to have a minimum of N = 3 biological replicates with sufficient reproducibility.
Data exclusions	No data were excluded from the analyses.
Replication	All experimental findings were replicated at least 3 times with enough reproducibility. Attempts of data replication were successful.
Randomization	Allocation of samples was random.
Blinding	For organoid experiment execution and data acquisition, researchers were blinded to sample allocation. For animal treatment experiments, investigators were not blinded to group allocation during data collection due to limited lab personnel.

Reporting for specific materials, systems and methods

We require information from authors about some types of materials, experimental systems and methods used in many studies. Here, indicate whether each material, system or method listed is relevant to your study. If you are not sure if a list item applies to your research, read the appropriate section before selecting a response.

Materials & experimental systems

n/a	Involved in the study	
[ ]	[x]	Antibodies
[ ]	[x]	Eukaryotic cell lines
[x]	[ ]	Palaeontology
[ ]	[x]	Animals and other organisms
[x]	[ ]	Human research participants
[x]	[ ]	Clinical data

Methods

n/a	Involved in the study	
[x]	[ ]	ChIP-seq
[ ]	[x]	Flow cytometry
[x]	[ ]	MRI-based neuroimaging

Antibodies

Antibodies used	For IF and IHC: Keratin 8 (#TROMA-1, Developmental Studies Hybridoma Bank), E-cadherin (#610182, BD Biosciences), and pan cytokeratin (#ab9377, Abcam).<br>For Immunoblotting: E-cadherin (1:1000, #3195, Cell Signaling Technology (CST)), Keratin 8 (1:1000, #TROMA-1, Developmental Studies Hybridoma Bank), Zeb1 (1:1000, #3396, CST), GAPDH (1:3000, #2118, CST), Slug (1:1000, #9585, CST), AcH3 (1:2000, #06-599, Upstate), and AcH4 (1:2000, #06-598, Upstate).
Validation	All the antibodies used in IF and IHC were widely cited. For example, Keratin 8, PMID: 26526198; E-cadherin, PMID: 11706048; pan-cytokeratin, PMID: 30664790.<br>Zeb1 and Slug antibodies used in immunoblotting assays have been validated by siRNA knockdown experiments. The data for these siRNA experiments were included in the uncropped blots file. E-cadherin, AcH3, and AcH4 antibodies used in immunoblotting assays have been validated by treating cells with drugs known to induce their expression levels. GAPDH antibody is widely cited, e.g. PMID: 32966795.

Eukaryotic cell lines

Policy information about cell lines

Cell line source(s)	Primary cell lines were generated from genetically modified mouse (GEM) mammary tumors. All the GEM models were heavily characterized by sequencing and histological studies.
Authentication	None of the cell lines used were authenticated.

Mycoplasma contamination	All cell lines tested negative for mycoplasma contamination.
Commonly misidentified lines (See ICLAC register)	No commonly misidentified cell lines were used in the study.

Animals and other organisms

Policy information about studies involving animals; ARRIVE guidelines recommended for reporting animal research

Laboratory animals	Mouse, BALB/c, Female, 6-8 weeks old
Wild animals	No wild animals were used in the study.
Field-collected samples	No field collected samples were used in the study.
Ethics oversight	This study was performed in accordance with the rules of the Guide for the Care and Use of Laboratory Animals of the NIH. All mice were maintained and killed according to guidelines of our institutional IACUC (Protocol AN-504).

Note that full information on the approval of the study protocol must also be provided in the manuscript.

Flow Cytometry

Plots

Confirm that:

- [x] The axis labels state the marker and fluorochrome used (e.g. CD4-FITC).
- [x] The axis scales are clearly visible. Include numbers along axes only for bottom left plot of group (a 'group' is an analysis of identical markers).
- [ ] All plots are contour plots with outliers or pseudocolor plots.
- [ ] A numerical value for number of cells or percentage (with statistics) is provided.

Methodology

Sample preparation	Cells were labelled with reporters and trypsinized to single cells for flow cytometry.
Instrument	Attune NXT
Software	Flowjo
Cell population abundance	No cell sorting was involved.
Gating strategy	Single cells were gated based on FSC-A versus FSC-H. All single cells were used for analyses.

- [x] Tick this box to confirm that a figure exemplifying the gating strategy is provided in the Supplementary Information.
