# Supplementary material for: Morphological screening of mesenchymal mammary tumor organoids to identify drugs that reverse epithelial-mesenchymal transition
Source: Nat Commun. 2021 Jul 12;12:4262. doi: 10.1038/s41467-021-24545-3 (PMC8275587; doi:10.1038/s41467-021-24545-3)
Supplement: Supplementary file 12 — Source Data [file 41467_2021_24545_MOESM12_ESM.zip › 283482_3_data_set_5643496_qddpt7.pptx]

## Slide 1
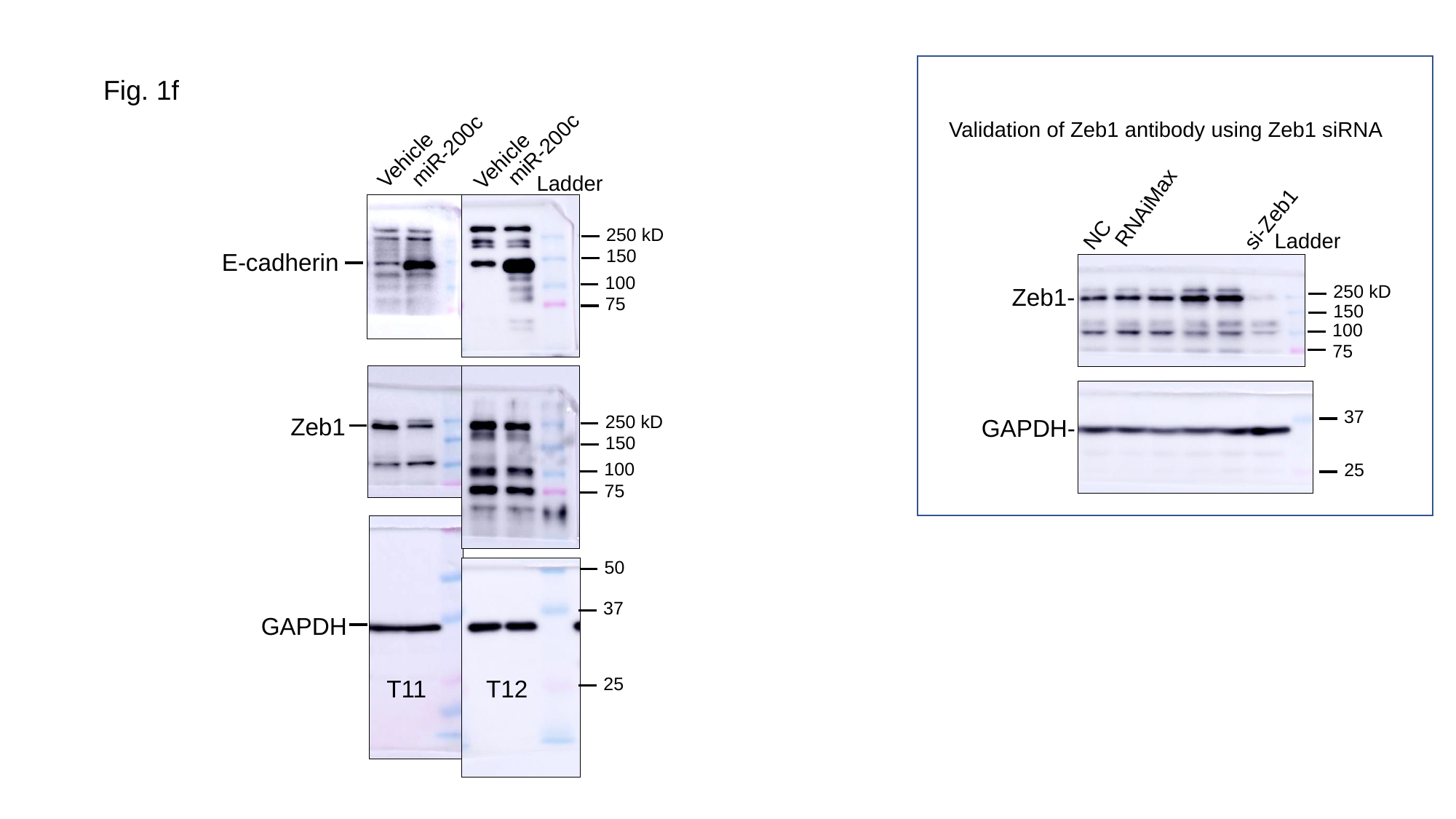

Fig. 1f
Validation of Zeb1 antibody using Zeb1 siRNA
miR-200c
miR-200c
Vehicle
Vehicle
Ladder
RNAiMax
si-Zeb1
NC
250 kD
Ladder
150
E-cadherin
100
250 kD
Zeb1-
75
150
100
75
37
250 kD
Zeb1
GAPDH-
150
100
25
75
50
37
GAPDH
25
T12
T11

## Slide 2
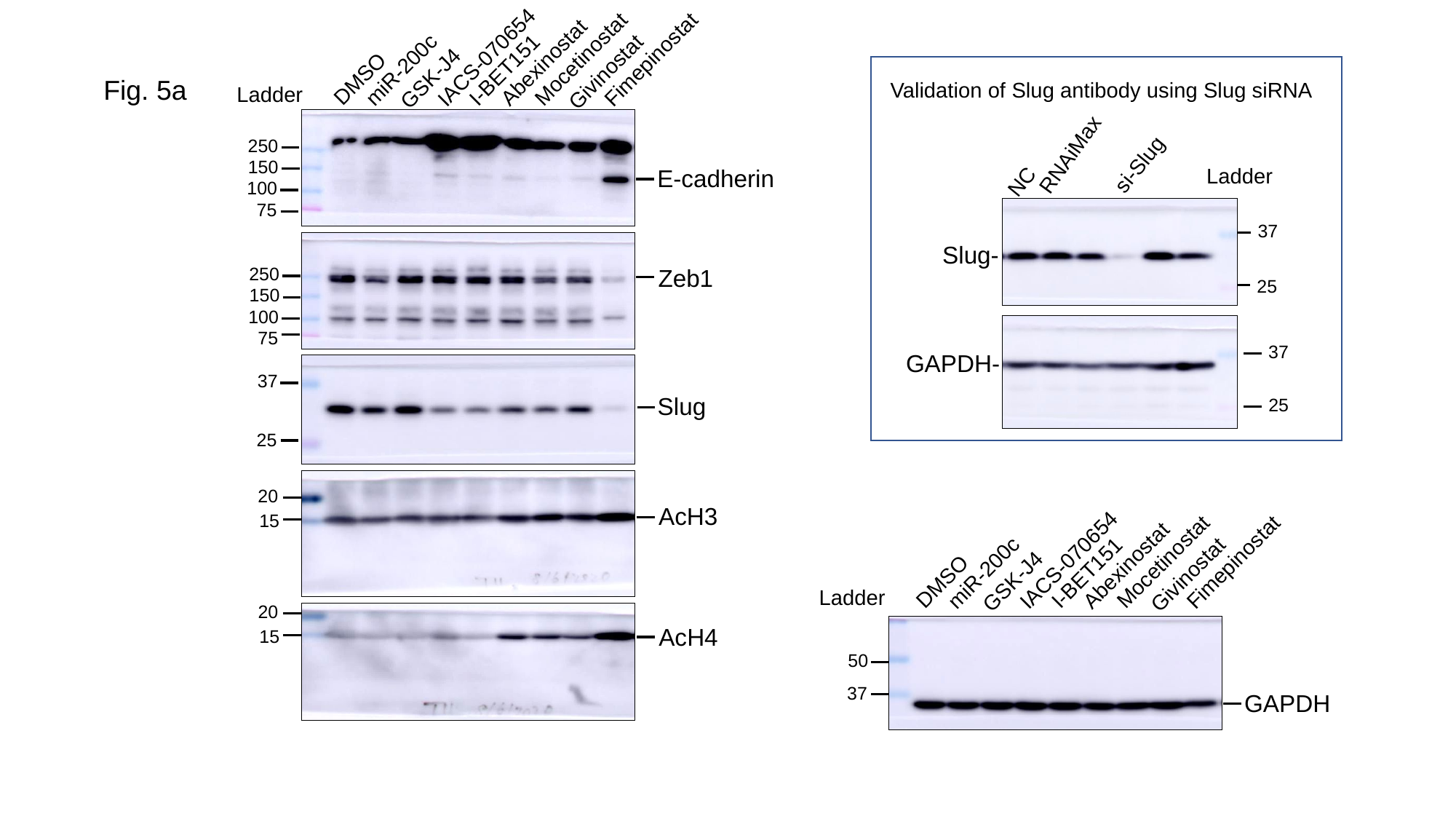

IACS-070654
Mocetinostat
Fimepinostat
Abexinostat
miR-200c
I-BET151
Givinostat
GSK-J4
DMSO
Fig. 5a
Validation of Slug antibody using Slug siRNA
Ladder
250
RNAiMax
si-Slug
150
Ladder
E-cadherin
NC
100
75
37
Slug-
250
Zeb1
25
150
100
75
37
GAPDH-
37
Slug
25
25
20
AcH3
15
IACS-070654
Mocetinostat
Fimepinostat
Abexinostat
miR-200c
I-BET151
Givinostat
GSK-J4
DMSO
Ladder
20
AcH4
15
50
37
GAPDH

## Slide 3
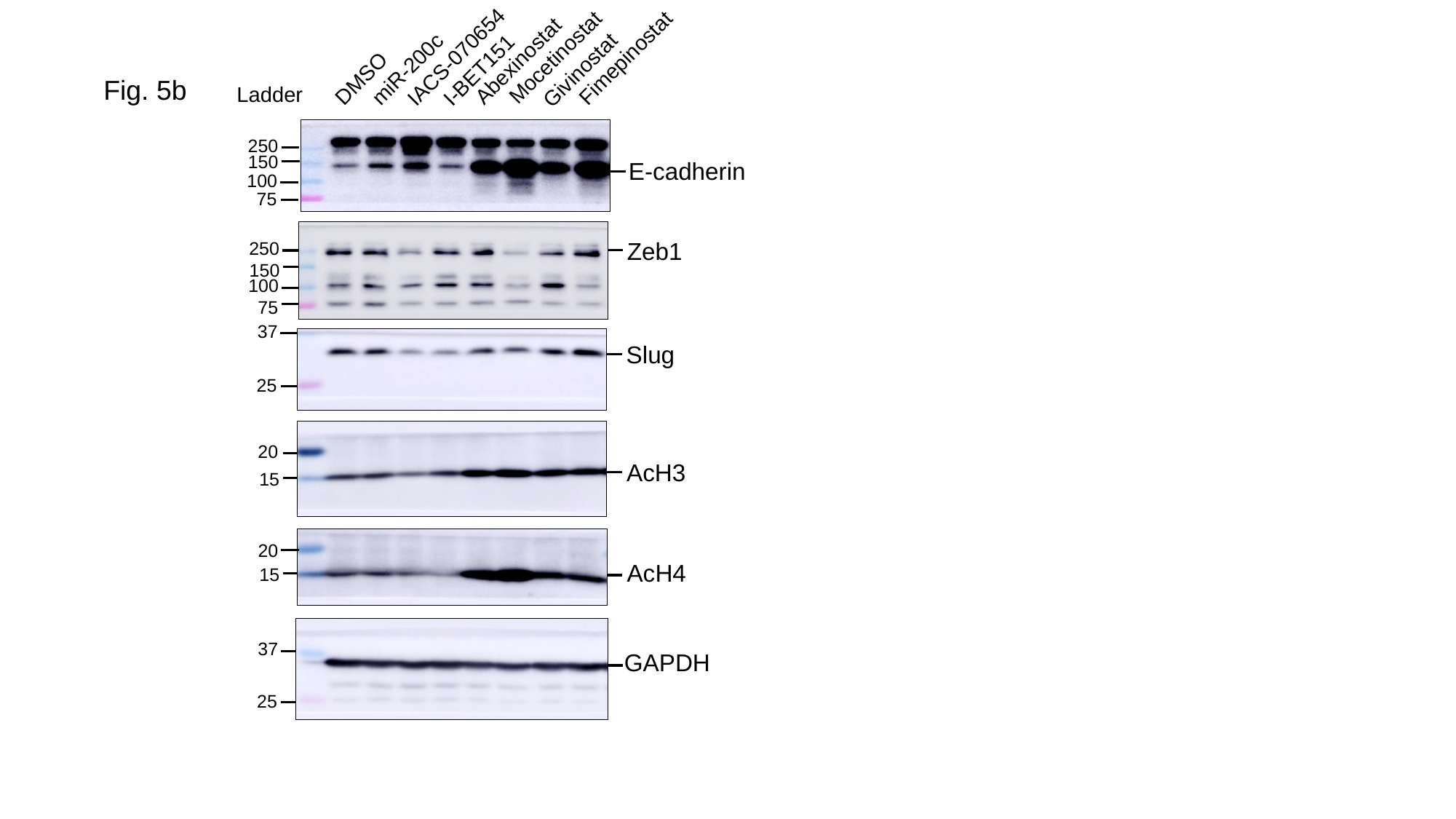

IACS-070654
Mocetinostat
Fimepinostat
Abexinostat
miR-200c
Givinostat
I-BET151
DMSO
Fig. 5b
Ladder
250
150
E-cadherin
100
75
Zeb1
250
150
100
75
37
Slug
25
20
AcH3
15
20
AcH4
15
37
GAPDH
25

## Slide 4
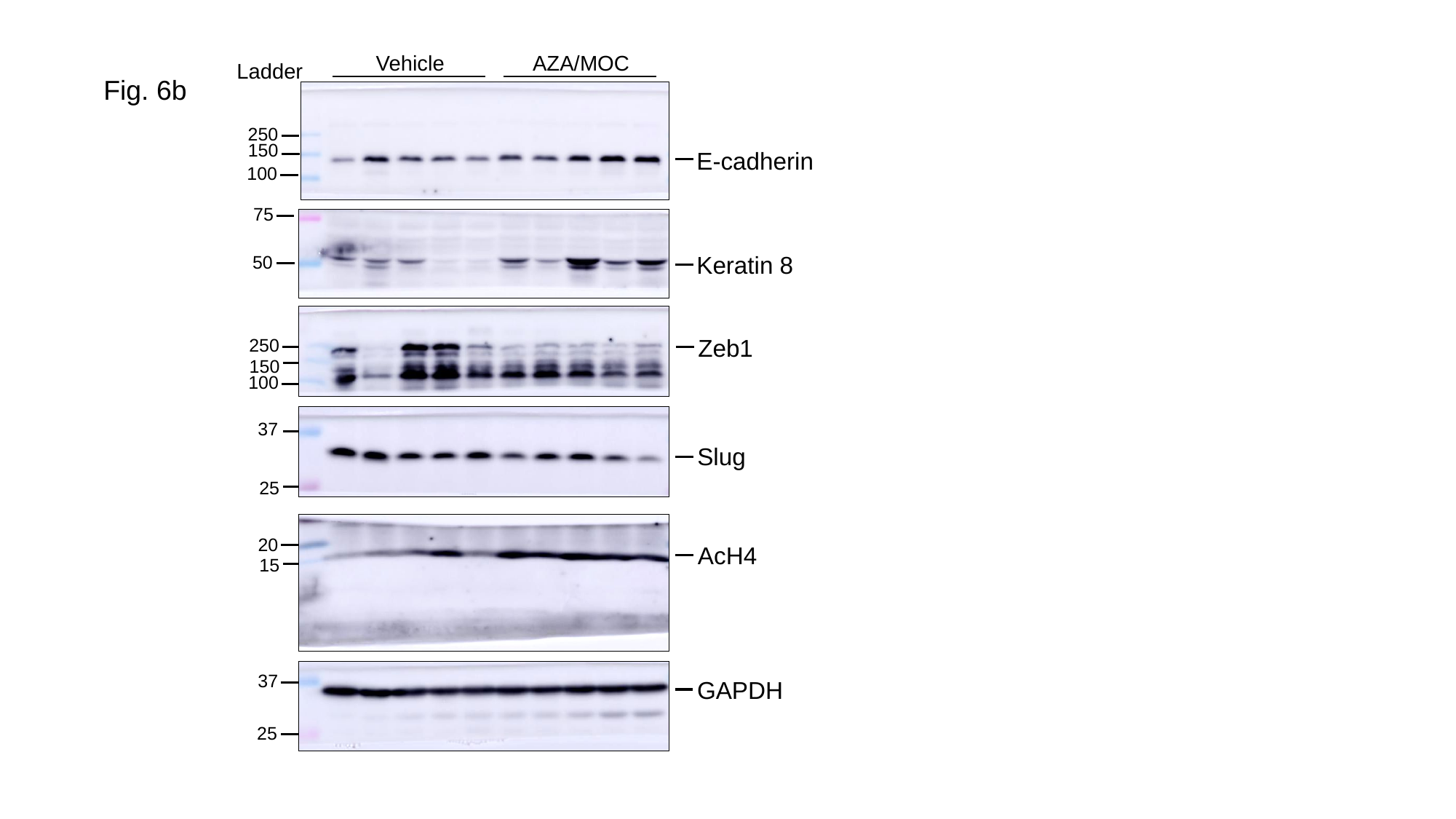

Vehicle
AZA/MOC
Ladder
Fig. 6b
250
150
E-cadherin
100
75
Keratin 8
50
Zeb1
250
150
100
37
Slug
25
20
AcH4
15
37
GAPDH
25

## Slide 5
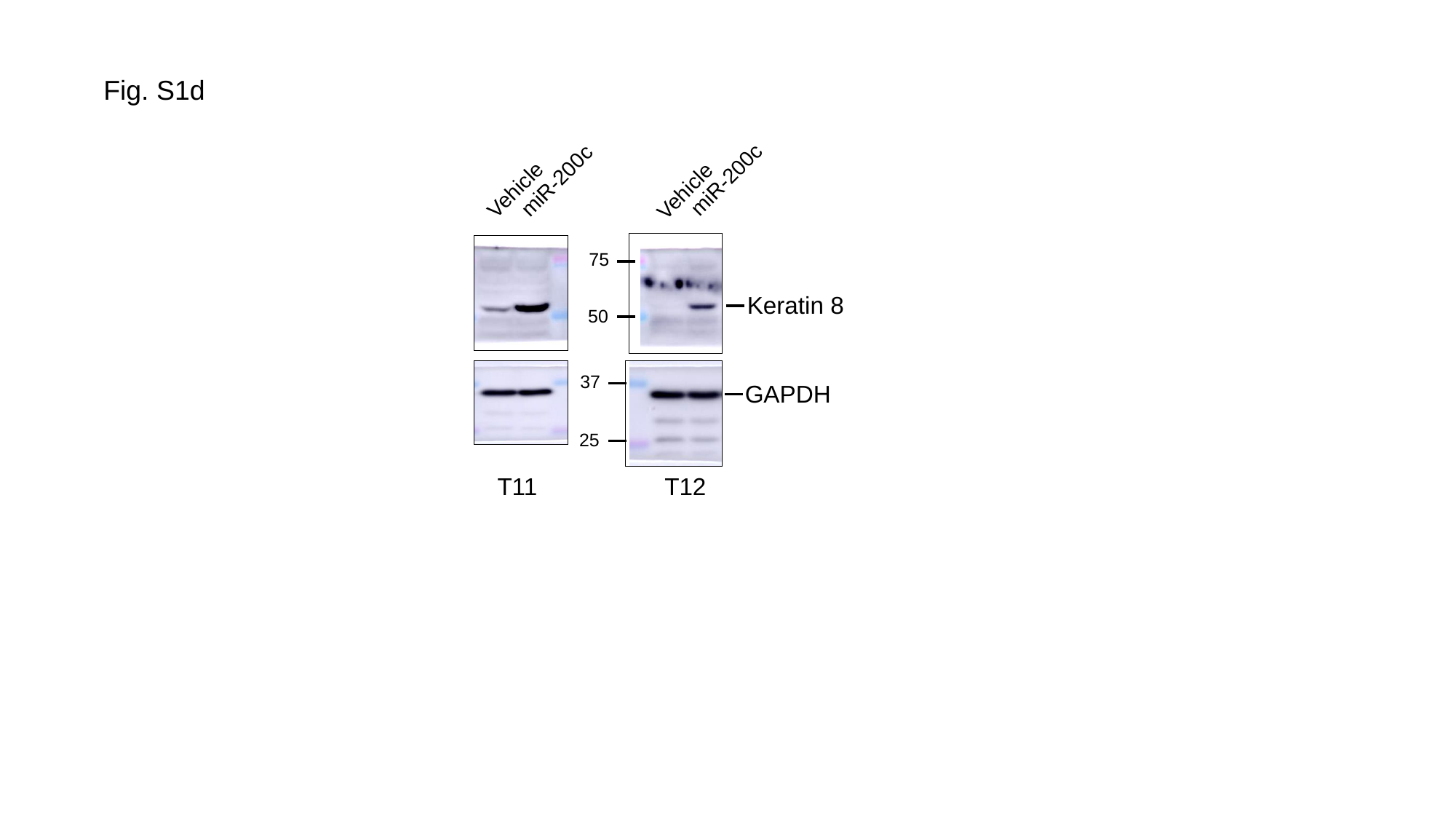

Fig. S1d
miR-200c
miR-200c
Vehicle
Vehicle
75
Keratin 8
50
37
GAPDH
25
T12
T11

## Slide 6
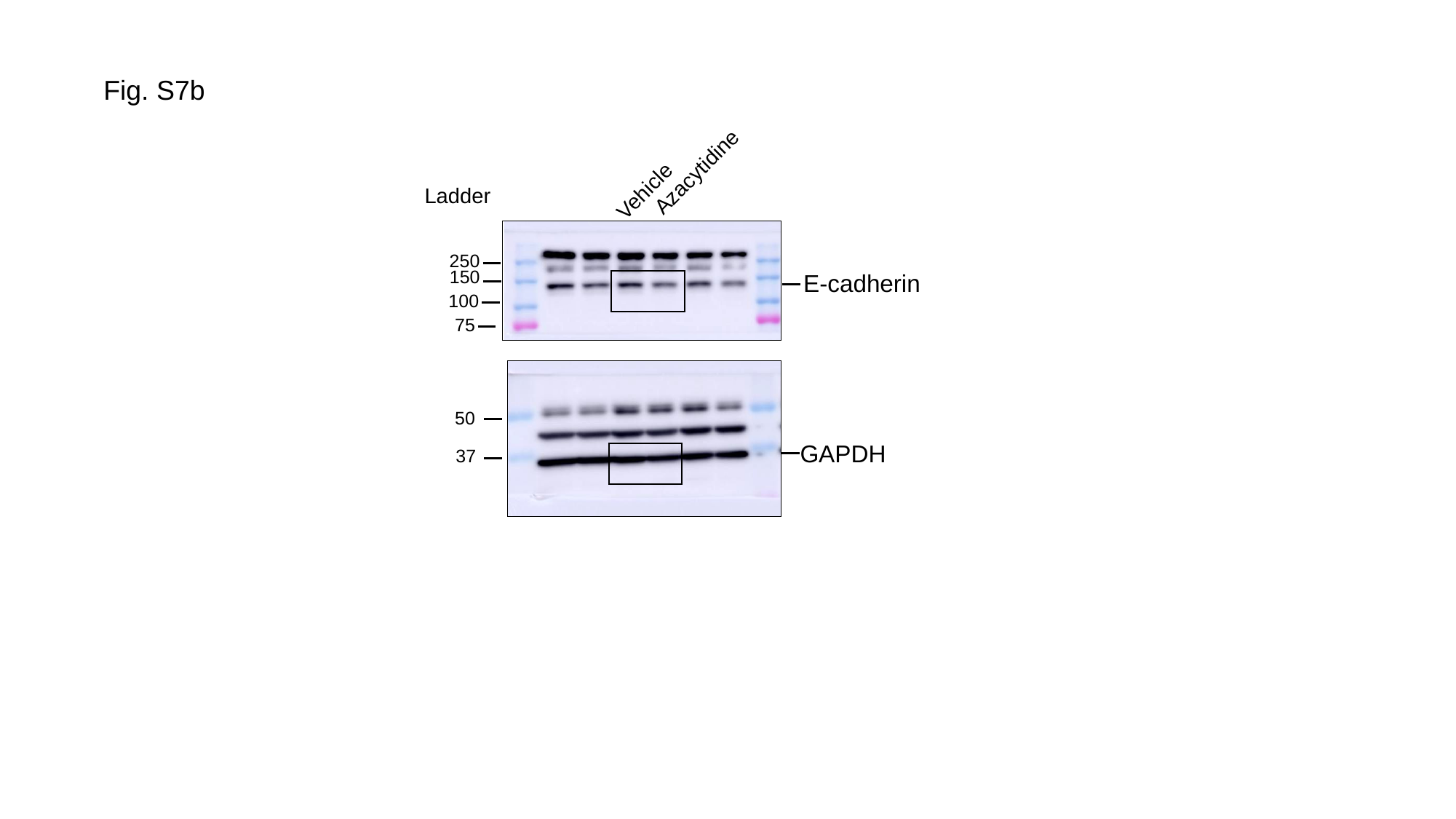

Fig. S7b
Azacytidine
Vehicle
Ladder
250
150
E-cadherin
100
75
50
GAPDH
37
